# Motivation, attention, and uncertainty: insights from animal and human research and implications for addiction

**DOI:** 10.3389/fpsyg.2026.1796424

**Published:** 2026-04-29

**Authors:** Michelle Heck, Etienne Quertemont, Patrick Anselme

**Affiliations:** 1 Psychology & Neuroscience of Cognition—PsyNCog, Université de Liège, Liège, Belgium; 2 Department of Biopsychology, Ruhr-University Bochum, Bochum, Germany

**Keywords:** addiction, attentional capture, incentive salience, motivation, reward, uncertainty

## Abstract

Motivational and attentional processes jointly determine how environmental cues influence behavior. Reward-predictive cues can acquire incentive salience, automatically capturing attention and eliciting approach responses even in the absence of deliberate intention, a phenomenon central to both adaptive behavior and certain forms of psychopathology. This review integrates animal and human evidence to examine how cue-triggered motivation and attention interact, and how these interactions are shaped by reward uncertainty, a dimension that has received limited interest in human research despite its robust effects in animals. Across species, uncertain or variably reinforced cues reliably intensify motivated behaviors. In animals, they enhance sign-tracking and promote persistent cue-directed responding, while in humans, paradigms such as uncertainty-modulated attentional capture (UMAC) show that uncertain cues exert a greater pull on attention, even when this impairs performance. We argue that these effects reflect an evolutionarily conserved mechanism that biases organisms toward exploration and information-seeking under unpredictable conditions. However, the same mechanisms can become maladaptive in modern environments, such as gambling or drug use, where artificially amplified reward signals can mimic or exaggerate the motivational impact of uncertainty, leading to compulsive cue attraction. By bridging motivational and attentional perspectives across animal and human research, this review offers a unified framework for understanding how reward cues gain motivational significance, how uncertainty modulates these processes, and why they may contribute to vulnerability in addiction-related behaviors.

## 1 Introduction

Environmental cues exert a powerful influence on everyday behavior. In a supermarket, for example, consumers may attend to specific products simply because a red or yellow price tag signals a discount, even before consciously evaluating the actual price. Likewise, the sound of a smartphone notification often elicits an almost automatic urge to check the device, as this auditory cue has become associated with rewarding social or informational content. Our understanding of such cue-driven behaviors originates from Pavlov's (1927) classical conditioning experiments, which demonstrated that a previously neutral stimulus can acquire the status of a conditioned stimulus (CS) when repeatedly paired with a reinforcing unconditioned stimulus (US), famously illustrated by dogs salivating at the sound of a bell preceding food delivery. Pavlov interpreted this phenomenon as a change in what the dogs learned about the association between the two stimuli. However, subsequent decades of animal research have revealed that conditioning also involves a shift in the organism's motivational state regarding the initially neutral stimulus: the CS not only predicts the timing of US delivery but also becomes imbued with the motivational salience of the US (Berridge and Robinson, 1998; Bindra, 1978; Toates, 1986). This capacity for cue-triggered behavior is highly conserved across the animal kingdom, from unicellular organisms (Carrasco-Pujante et al., 2021) to “higher” vertebrates, including humans.

Under some conditions, cue-triggered responses can become excessively automatic, rigid, and misaligned with an individual's broader goals. These characteristics are particularly evident in addictive behaviors and have been extensively investigated in laboratory animals. In the context of drug addiction, cue reactivity plays a pivotal role in both the persistence of addictive behavior and the heightened risk of relapse among abstinent individuals (Everitt et al., 2001; Robinson and Berridge, 1993; Wiers and Stacy, 2006; Witteman et al., 2015). This incentive process is largely unconscious and cue-driven, as it requires neither deliberate choice nor cognitive inference (Robinson and Berridge, 2025). The capacity of reward-associated cues to capture attention and enhance incentive salience is not limited to drugs of abuse. Attentional bias toward reward-paired cues has been documented across various reward domains, including food (e.g., Deluchi et al., 2017; Field et al., 2016; Yokum et al., 2011) and sexual stimuli (e.g., Mechelmans et al., 2014; Sklenarik et al., 2020).

Addictive-like effects are also observed in gambling and gambling-like contexts, which involve mere exposure to reward uncertainty, i.e., when predictive cues are followed by a reward or its omission in a random manner. For instance, rats exhibit stronger responses to a CS that inconsistently predicts food compared to a CS that consistently predicts food (Anselme et al., 2013; Glueck et al., 2018; Robinson et al., 2023), an effect documented across other species as well (Crawford et al., 1985; Gottlieb, 2004). Notably, reward uncertainty seems to induce long-lasting increases in cue responsiveness, persisting even after uncertainty diminishes or disappears (Crawford et al., 1985; Robinson M. J. F. et al., 2014). In humans, casinos exemplify gambling environments saturated with cues, such as flashing lights and celebratory sounds, that are repeatedly presented both prior to and during reward delivery (Griffiths, 1993; Noseworthy and Finlay, 2009; Parke and Griffiths, 2006). Over time, these cues can acquire excessive attractiveness and motivate individuals to continue gambling (Barrus and Winstanley, 2016; Brevers et al., 2014; Dixon et al., 2014).

This review proposes an integrative view of how motivation, attention, and uncertainty interact in a context of reward-predictive cues influencing motivated behavior in human and nonhuman animals. We examine how incentive motivation and attentional priority interact to shape cue-driven behavior, with a particular focus on the role of reward uncertainty, a dimension that has surprisingly been poorly investigated in the human literature despite its robust effects in animal research. In this article, we focus specifically on cue-related uncertainty in situations in which uncertainty is expected and stable over time, as is typical in most laboratory conditions. Situations in which uncertainty is unexpected, fluctuates over time (volatility) or is transient (such as during extinction or reversal learning) may involve overlapping processes but will not be discussed here (for details, see Soltani and Izquierdo, 2019). Risk, as a form of uncertainty typically distinguished from ambiguity, is also excluded. In the present review, we are interested in the incentive, often unconscious and automatic, effects that reward uncertainty exerts on behavior, even though uncertainty-sensitive neurons are also found in cortical regions traditionally associated with higher-order cognitive functions, such as the anterior cingulate cortex and the orbitofrontal cortex (e.g., Karlsson et al., 2012; Monosov, 2020; Romero-Sosa et al., 2025). The incentive and addictive effects of reward uncertainty are likely to be more directly controlled by subcortical regions such as the ventral tegmental area and nucleus accumbens (Singer et al., 2020). By bringing together findings from animal models and human paradigms, we highlight how uncertainty can amplify the impact of reward-associated cues and fundamentally alter their processing. Table 1 summarizes converging findings from both animal and human studies on the effects of uncertainty, alongside the key theoretical explanations proposed in the literature and discussed throughout this paper. Finally, we consider the functional significance of these uncertainty-driven effects, emphasizing their adaptive role in exploration and information-seeking, as well as their potential to become maladaptive in modern contexts such as gambling and addiction.

**Table 1:** Comparative summary of uncertainty-driven modulation of cue-guided behavior in rodents and humans.

Context	Population	Effect	Proposed explanations
Pavlovian conditioned approach behavior	Non-human animals	↑ Sign-tracking and ↑ DA release in sign-trackers	• Reward uncertainty continuously signals that learning is incomplete, thereby maintaining elevated mesolimbic dopamine levels (Schultz et al., 1993). • Uncertainty-driven behavior mostly results from effort mobilization via HPA axis and locus coeruleus to increase success rate (Anselme, 2025). • Uncertainty directly contributes to information- and novelty-seeking, meaning the strategy may disambiguate context and reduce uncertainty (Bromberg-Martin and Monosov, 2020; Monosov, 2024).
Early, prolonged uncertainty exposure in pavlovian tasks	Non-human animals	↑ Sensitization process (related to IST)	• Early exposure to uncertainty might produce lasting behavioral sensitization, with effects resembling drugs of abuse and gambling-like tasks (Mascia et al., 2019; Robinson et al., 2015; Singer et al., 2012; Swintosky et al., 2021; Zack et al., 2014). • Addictive drugs may artificially trigger phasic dopamine surges mimicking motivational amplifications observed under unpredictable reward conditions.
UMAC	Humans	↑ Attentional capture by “high uncertainty” cues	• Cues with high uncertainty attract greater attention because their consequences potentially require increased monitoring (Uncertainty Principle; Pearce and Hall, 1980). • Parallel suggested between uncertainty-driven attentional capture and exploration (Cho and Cho, 2021; Chow et al., 2025; Ju and Cho, 2023; Le Pelley et al., 2019; Pearson et al., 2024). Uncertainty-related attentional capture may reflect a functional mechanism to update internal models of reward contingencies rather than maladaptive distraction.

## 2 Animal research

### 2.1 From incentive motivation to addiction

**Figure 1:**
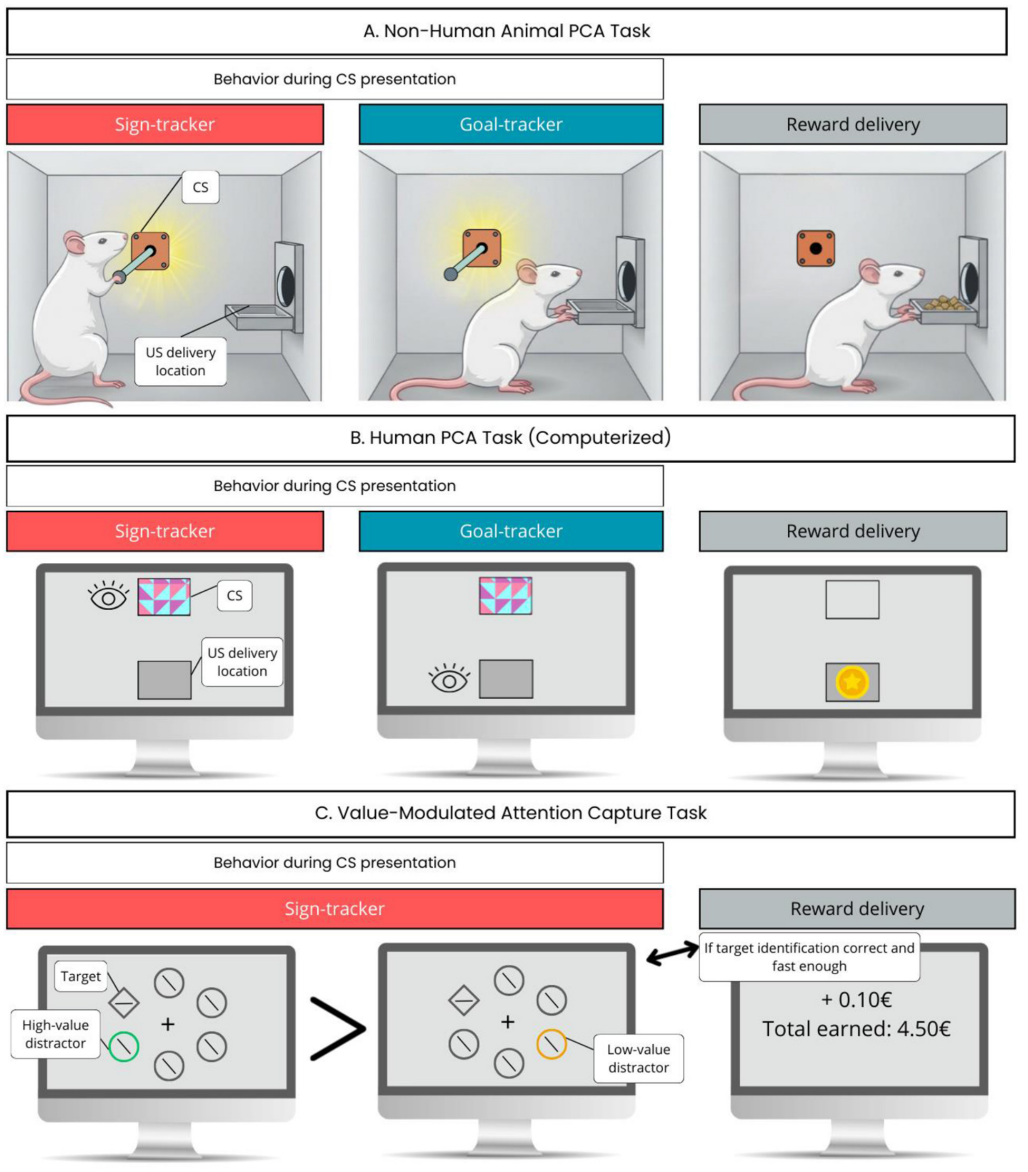
**(A)** Rodent PCA task. The first panel illustrates characteristic sign- and goal-tracking behavior in a rodent PCA task. During CS presentation, ST animals orient toward the retractable lever and may approach, touch, or gnaw it. In contrast, GT animals orient toward the location of impending reward delivery (the food tray). **(B)** Human PCA task. The second panel illustrates analogous human ST and GT behaviors in a computerized PCA paradigm. While the CS (e.g., a fractal image displayed at the top of the screen) is presented, ST individuals show increased dwell time on the CS itself, whereas GT individuals allocate more gaze time toward the goal location where the reward will subsequently appear (e.g., a coin icon). **(C)** VMAC task. The third panel illustrates sign-tracking-like behavior in a VMAC task. Here, ST tendencies manifest as prolonged reaction times when the search display includes a high-value reward-predicting stimulus relative to a low-value CS. Although this task does not provide a direct behavioral analog of goal-tracking, sign-tracking is generally considered a continuum; thus, reduced sign-tracking-like effects may be interpreted as more GT-like behavior.

Before examining how expected reward uncertainty alters behavior, we first provide a brief overview of incentive motivational processes (Section 2.1) and their interaction with attentional mechanisms (Section 2.2). Indeed, these two dimensions are consistently highlighted as key determinants of basic reactivity to this form of uncertainty in the animal literature (Section 2.3). In Pavlov's (1927) seminal experiments on CS–US associations in dogs, the animals were restrained during measurements because Pavlov's primary interest concerned physiological responses, such as salivation and gastric secretion, under tightly controlled conditions. Had the dogs been free to move, Pavlov might have observed some individuals approaching the ringing bell prior to food delivery. Decades later, such cue-triggered approach behavior was termed sign-tracking (Hearst and Jenkins, 1974) and is now understood to reflect not only associative learning but also incentive motivation, conceptualized through the notion of incentive salience or “wanting” (Berridge and Robinson, 1998). In other words, sign-trackers do not merely recognize the CS as a predictor of reward; they attribute to it a motivational value that makes the cue attractive and desirable in itself. The experimental paradigm that revealed sign-tracking, and remains widely used, is Pavlovian autoshaping. In this procedure, each trial consists of presenting a CS (typically a metal lever for rats) for a few seconds, followed immediately by the delivery of food pellets into a dish. Training sessions include multiple trials separated by inter-trial intervals during which no events occur. Importantly, no instrumental action is required for reward delivery. Apart from sign-trackers that approach and interact with (pressing, sniffing, nibbling, biting) the lever CS due to its acquired motivational properties (Flagel et al., 2007; Robinson and Flagel, 2009), other individuals, called goal-trackers, instead approach and inspect the food dish during CS presentation, anticipating reward delivery (Boakes, 1977). A third group exhibits an ambivalent profile, alternating between sign- and goal-tracking behaviors. Figure 1A illustrates sign- and goal-tracking behaviors in a typical animal PCA task (autoshaping).

Interestingly, sign- and goal-trackers exhibit distinct neurobiological signatures in response to the CS, particularly regarding mesolimbic dopamine release from the ventral tegmental area to the nucleus accumbens. Well-trained sign-trackers display a pronounced phasic dopamine surge at CS presentation, which drives approach toward the cue, whereas goal-trackers show little or no dopamine release beyond baseline levels at this time (Flagel et al., 2011b). This dissociation is especially striking given that both phenotypes acquire the CS–US association with comparable efficiency (Meyer et al., 2012). These observations, among others, strongly support the incentive salience theory, which posits that dopamine confers motivational attractiveness to reward-predictive cues (e.g., Day et al., 2006; Robinson and Berridge, 2013; Saunders and Robinson, 2012). Experimental manipulations of dopamine release further demonstrate its role in “wanting”: altering dopamine release changes the propensity to approach a cue without affecting hedonic reactions to the reward (e.g., Flagel et al., 2011b; Peciña et al., 2003). Similarly, dopamine-deficient mice fail to attribute incentive salience to food-related stimuli. They do not initiate approach behavior and would starve even when surrounded by palatable food, due to a genetic inability to synthesize dopamine. Daily L-Dopa administration is required to restore feeding behavior (Palmiter, 2008). Remarkably, these deficits are not due to motor impairments: dopamine-deficient mice readily climb cage walls and traverse widely spaced metal grids without difficulty. Moreover, when food intake is assisted, they consume normal amounts and exhibit intact hedonic preferences, favoring sucrose over less palatable options (Cannon and Bseikri, 2004; Cannon and Palmiter, 2003).

An alternative interpretation of dopamine's role in this context is that it encodes reward prediction error (RPE), a mechanism fundamental to associative learning (Schultz, 1998; Starkweather and Uchida, 2021). Under this account, phasic dopamine responses initially occur at reward (US) delivery and progressively shift to the CS as learning unfolds, such that dopamine responses to the CS reflect the predictive value of the cue (Schultz et al., 1997; Tobler et al., 2006). Although this shift is robustly observed in sign-trackers, it is noticeably absent in goal-trackers, which exhibit sustained dopamine responses to the US across training despite having learned the CS–US contingency (Flagel et al., 2011b). This pattern challenges a pure RPE-based interpretation, as both phenotypes should exhibit similar dopaminergic dynamics, if dopamine is limited to encode prediction error. Pharmacological and circuit-level manipulations further dissociate predictive learning from incentive salience. For example, systemic or local blockade of dopamine receptors, lesions of the nucleus accumbens, or temporally precise optogenetic inhibition of ventral tegmental dopamine neurons during CS presentation selectively disrupt the acquisition of sign-tracking while sparing goal-tracking behavior (Chow et al., 2016; Flagel et al., 2011b; Iglesias et al., 2023; Saunders and Robinson, 2012).

These findings align more closely with the incentive salience theory, according to which dopamine release reflects the attribution of motivational properties to the CS rather than its predictive value (Robinson and Flagel, 2009). Consistent with this view, an experimentally induced increase in dopamine can instantly transform a strongly aversive CS into an intensely “wanted” stimulus without requiring relearning (e.g., Robinson and Berridge, 2013; Tindell et al., 2009). Integrative models were proposed to reconcile incentive salience and RPE accounts, suggesting that dopaminergic RPE signals mediate incentive salience attribution, with individual differences reflecting the dominance of distinct learning systems (Dayan and Berridge, 2014; Flagel et al., 2011b; Huys et al., 2014; Schad et al., 2020). Within this framework, sign-trackers rely predominantly on dopamine-dependent, model-free learning processes, whereas goal-trackers depend more on model-based learning centered on state prediction errors (SPEs). Consistent with this interpretation, behavioral and computational studies show that sign-tracking is associated with stronger model-free reinforcement learning across both Pavlovian and instrumental domains (Moin Afshar et al., 2023). Furthermore, recent computational modeling suggests that, during the transfer phase of a Pavlovian-to-instrumental transfer task, sign-trackers exhibit slower updating of Pavlovian cue values rather than an overweighting of Pavlovian values relative to instrumental action values (Degni et al., 2026). Collectively, these behavioral, neurobiological, and computational findings converge on the conclusion that dopamine-dependent model-free learning underlies incentive salience attribution, providing a coherent account of individual differences between sign- and goal-trackers.

The attribution of incentive salience to a cue does not require any conscious awareness; this process has been suggested to originate in deep brain structures, largely independent of cortical influences and phenomenal consciousness (Berridge, 2003; Winkielman and Berridge, 2003). Unlike desires, which can be pursued or inhibited based on circumstances due to their goal-directed and cognitive properties, incentive salience may drive the irrational pursuit of cues, even at the expense of physical and mental health. Consistent with this view, animal research has documented the role of incentive salience in addiction. For instance, numerous studies report that sign-trackers, that attribute high incentive salience to reward-predictive cues, are more vulnerable to drugs of abuse than goal-trackers (Flagel et al., 2010; Saunders and Robinson, 2011; Tomie et al., 2008).

Sign-trackers and goal-trackers differ markedly in their sensitivity to key features of addiction. Sign-trackers exhibit reduced behavioral flexibility and stronger cue-driven motivation compared to goal-trackers. For example, they persist in approaching the CS even when the associated reward becomes less palatable (reward devaluation; Morrison et al., 2015) or when the reward is no longer delivered (extinction; Ahrens et al., 2016). In contrast, goal-trackers adjust their behavior more readily to changing contingencies (Pellón et al., 2018; Strand et al., 2022). Persistence despite negative outcomes is a hallmark of addictive behavior. Accordingly, sign-trackers display a greater propensity to self-administer drugs and to prefer drugs over food rewards compared to goal-trackers (Tunstall and Kearns, 2015; Versaggi et al., 2016). Furthermore, they are more prone to relapse when exposed to cues previously associated with drug consumption (Saunders et al., 2013; Versaggi et al., 2016; Yager et al., 2015). Beyond these behavioral outcomes, sign-trackers differ from goal-trackers in what has been described as a “constellation of cognitive–motivational characteristics” that confers heightened vulnerability to continued drug use following initial exposure (Flagel et al., 2021; Robinson et al., 2018). Specifically, sign-trackers tend to be more impulsive (Lovic et al., 2011; Tomie et al., 1998) and to exhibit weaker top-down executive control (Enkel et al., 2019; Koshy Cherian et al., 2017; Paolone et al., 2013), traits that have been systematically associated with substance use and addiction in humans (Lejuez et al., 2010; Verdejo-Garcia and Albein-Urios, 2021). Once drug use becomes established, drugs of abuse exert their addictive potential primarily through the dopaminergic system. They act as powerful activators of mesolimbic dopamine pathways and, with repeated exposure, induce enduring neurochemical changes that sensitize dopamine neurons (Robinson and Berridge, 1993, 2025). This process, known as incentive sensitization, represents a pathological overactivation of the “wanting” system. Unlike normal motivational states (e.g., hunger, sex, play), which are transient and regulated by homeostatic mechanisms, drug-induced sensitization is persistent and leads to exaggerated dopamine responses in mesocorticolimbic regions, particularly the nucleus accumbens. This enduring change explains why drug-associated cues remain highly motivational and can trigger craving and relapse long after withdrawal symptoms have subsided (Castner and Goldman-Rakic, 1999; Paulson and Robinson, 1995). Taken together, these findings suggest that sign-trackers are uniquely vulnerable to addiction due to their heightened tendency to attribute incentive salience to reward-predictive cues combined with a cognitive-motivational profile characterized by impulsivity and diminished executive control. This vulnerability increases the likelihood of drug initiation, facilitates the maintenance of drug use, and heightens the risk of cue-induced relapse. Moreover, with prolonged drug exposure, incentive sensitization processes further amplify cue reactivity (but importantly, not only in sign-trackers but also in goal-trackers, see Kawa et al., 2016).

### 2.2 Interaction between incentive motivation and attention: dopamine–acetylcholine dynamics

Motivation and attention often operate in tandem. Greater motivation for a stimulus typically biases attention toward that stimulus. In Pavlovian autoshaping, sign-trackers approach the CS while goal-trackers approach the food dish, suggesting that each phenotype allocates more attention to its preferred cue. But does this difference in preference translate into differences in attentional performance? A key neurochemical correlate of attentional performance is cortical acetylcholine, a neurotransmitter known to interact with dopaminergic mechanisms underlying incentive motivation (Sarter et al., 2006; Sarter and Bruno, 1999). Paolone et al. (2013) examined rats performing a sustained attention task and reported fluctuations between periods of good and poor performance, as well as automatic attraction to salient stimuli, in sign-trackers compared to goal-trackers (but see Kindedji and Huppé-Gourgues, 2024; Thériault et al., 2025). The poorer performance of sign-trackers was attributed to lower extracellular levels of cortical acetylcholine, a finding consistent with evidence for reduced top-down (cortical, cognitive) control over behavior in these individuals relative to goal-trackers (Flagel et al., 2011a). Differences in acetylcholine levels emerged during attentional tasks, although baseline levels were similar across these phenotypes (Sarter and Phillips, 2018). Experimental manipulations further support this interpretation: cholinergic agonists improve sign-trackers' performance, whereas antagonists can prevent the development of goal-tracking behavior (Koshy Cherian et al., 2017; Sarter and Phillips, 2018).

In summary, sign-trackers exhibit higher mesolimbic dopamine activity and lower cortical acetylcholine activity compared to goal-trackers, indicating that these phenotypes differ neurobiologically in how they process the “meaning” of the CS. When a CS is detected, the weak top-down regulation of dopamine by cortical acetylcholine in sign-trackers likely promotes a bias toward stimulus-driven (bottom-up) attention, facilitating progressive CS attraction through incentive salience. For these individuals, the CS becomes a potent incentive stimulus. In contrast, CS detection in goal-trackers increases acetylcholine without altering dopamine levels. This top-down cholinergic control inhibits dopamine neurons, supporting the formation of expectations and the expression of goal-directed behavior. Consequently, the CS functions as an informational signal predicting imminent reward rather than as an attractive object (Flagel et al., 2011a; Pitchers et al., 2017). Interestingly, however, the stronger attentional control of goal-trackers in Pavlovian tasks does not fully protect them from drug-related vulnerability. Robinson T. E. et al. (2014) reported that goal-trackers are more sensitive to contextual cues than to localized discrete cues. Similarly, Pitchers et al. (2017) found that discriminative stimuli, signaling drug availability without direct association with drug delivery, induce stronger relapse in goal-trackers than in sign-trackers (see also Ndiaye et al., 2024). Finally, following intermittent access to cocaine self-administration, behavioral differences between sign- and goal-trackers largely disappear (Kawa et al., 2016).

### 2.3 The stimulating effects of reward uncertainty

The role of uncertainty in modulating cue-driven behavior is both striking and theoretically puzzling. While the incentive salience theory provides a robust account of how reward-predictive cues become attractive, it does not explicitly address why unpredictability should enhance this effect. Yet, uncertainty often emerges as a powerful modulator of conditioned approach behavior, enhancing the motivational value of cues far beyond what their predictive value alone would suggest (e.g., Anselme et al., 2013; Glueck et al., 2018; Hellberg et al., 2019; Kaneko et al., 2025).

In most autoshaping studies, animals are trained under conditions of reward certainty, where each CS reliably predicts food delivery. However, when reward delivery becomes uncertain, for example with only half of the trials randomly rewarded, making each CS presentation only 50% predictive, a remarkable shift occurs. Animals exposed to this inconsistent CS–US association typically exhibit stronger and more persistent sign-tracking behavior than those trained with a fully predictive CS. This phenomenon, known as the partial reinforcement acquisition effect, is now well documented across species (e.g., Anselme et al., 2013; Crawford et al., 1985; Gottlieb, 2004; Pearce et al., 1985; Robinson M. J. F. et al., 2014).

This behavioral effect is robust, yet its theoretical basis remains poorly understood. It seems to contradict the incentive salience theory, which does not explicitly incorporate reward probability and would rather predict the opposite pattern, i.e., a decrease in sign-tracking when reward likelihood is lower. According to the incentive salience theory, the CS acquires motivational value proportional to the reward, which should diminish under uncertainty (Anselme, 2025; see Zhang et al., 2009; for a mathematical model of incentive salience). One might argue that uncertain rewards are inherently valued more than certain ones (Kahneman, 2011), but such claims extend beyond the original model and require additional mechanisms not specified in its original formulation (Anselme, 2025). Interestingly, goal-trackers exhibit the reverse pattern: their tendency to approach the food dish decreases under uncertainty (Gottlieb, 2005; Harris and Carpenter, 2011; Navarro et al., 2024). Notably, this reduction in goal-tracking was observed in conditions where animals had limited opportunities to interact with the inconsistent CS, which was presented as a light or tone. When the CS is a lever, both manipulable and retractable, a substantial proportion of individuals shift toward a sign-tracking profile and increase their response rates to the cue under reward uncertainty compared to reward certainty (Robinson et al., 2015). Beyond probabilistic uncertainty, temporal uncertainty can also enhance sign-tracking behaviors. Studies manipulating the inter-trial interval (ITI) show that animals display stronger cue-directed behavior when the ITI varies unpredictably than when it is fixed (Kaneko et al., 2025). Also, a longer ITI introduces unpredictability in the expected timing of both the CS and the US, making it difficult for animals to anticipate when reward-related events will occur (Gibbon et al., 1980; Lee et al., 2018; Mahmoudi et al., 2023).

In addition to increasing their response rates to the CS, sign-trackers exhibit greater dopamine release during uncertainty rather than certainty training (Hart et al., 2015). If dopamine is considered a marker of incentive motivation, this finding presents a conceptual puzzle: why should uncertainty act as an enhanced appetitive signal? Uncertainty reduces the predictive value of the CS, making it less attractive than a certain CS due to a lower transferability of the reward properties to the CS. Consistent with this, when animals are given a choice, they almost never prefer uncertain rewards over certain ones (de Jonge et al., 2008; Eisenreich et al., 2019; Gneezy et al., 2006; Kahneman, 2011). Therefore, uncertainty appears more aversive than appetitive, like a challenge to overcome rather than a reward cue to pursue (Anselme, 2025). This interpretation suggests that the dopaminergic surge observed under uncertainty may not reflect incentive salience. Instead, cue inconsistency could signal a difficulty that mobilizes effort-related systems, such as glucocorticoids via the hypothalamo–pituitary–adrenal (HPA) axis and noradrenaline via the locus coeruleus, two biological systems not directly involved in incentive salience attribution (Lopez et al., 2021; Varazzani et al., 2015). However, moderate glucocorticoid release is known to enhance dopamine activity (Lemos et al., 2012), making the observed dopamine increase partly an indirect effect of stress-related mechanisms. In this view, uncertainty-driven behavior may result from the combined influence of incentive salience and effort mobilization, rather than from cue attractiveness alone.

Taken together, these findings suggest that uncertainty-driven invigoration cannot be attributed to a single mechanism. Short-term effects likely arise from the combined influence of stress-related effort mobilization and heightened dopaminergic activity, whereas longer-term persistence may reflect gradual incentive sensitization. These processes also interact. When cues are unreliable, stress-related systems energize behavior and indirectly enhance dopamine release, such that repeated exposure to uncertainty progressively sensitizes dopaminergic pathways and sustains cue attraction over time. In pathological gamblers, this compensatory stress response appears to collapse (Biback and Zack, 2015), yet the dopaminergic sensitization produced by chronic exposure to inconsistent cues persists (van Holst et al., 2018) rendering these cues particularly compelling and difficult to resist.

An alternative explanation for the link between reward uncertainty and dopamine release can be found in RPE theory (Schultz, 1998). We reported earlier that with repeated exposure to a CS–US association, the predictive value of the CS increases, and the dopamine signal gradually shifts from the US to the CS until the US response disappears, becoming indistinguishable from baseline activity (Mirenowicz and Schultz, 1994). However, dopamine continues to be released even after extensive training when the interval between CS and US is randomly variable, because the association is impossible to learn (Schultz et al., 1993). In other words, reward uncertainty continuously signals that learning is incomplete, thereby maintaining elevated mesolimbic dopamine levels. Preuschoff and Bossaerts (2007) suggested that dopamine release does not only serve learning but also the ability to choose a more rewarding option, compared to a poorer one, as well as to track prediction risk, i.e., the scaling of prediction errors by means of the covariance between predictions and prior prediction errors. The mathematics behind this view captures how reward uncertainty influences dopamine release, as well as some aspects of the decision to prefer more potent rewards, which may be relevant to addictive processes. However, interpreting dopamine release as a predictive signal has limitations: it does not explain the direction or intensity of behavior in the absence of a motivational component (Anselme, 2016), whether the task requires instrumental action or Pavlovian responses such as sign-tracking. Also, there are situations in which motivation can be detached from prediction, that is, where intense “wanting” occurs despite prediction of a bad outcome (Berridge, 2023). For example, salt-deprived rats become instantly attracted by a lever predictive of the delivery of salt water in their mouth, after learning the aversive effect of this event in the absence of salt deprivation (Robinson and Berridge, 2013). Similarly, rats develop strong “wanting” about a metal rod predictive of electric shock, if approaching the rod is associated with optogenetic stimulation of central amygdala (Warlow et al., 2020).

Nevertheless, the RPE framework enables a functional perspective suggesting that behavioral invigoration under unavoidable reward uncertainty may reflect adaptive exploration rather than an appetitive response or a simple learning deficit. Beyond its role as a teaching signal, RPE can redirect attention toward the broader context when ambiguity arises (Rosas et al., 2006), a mechanism that facilitates uncertainty resolution in natural environments. In this perspective, uncertainty directly contributes to information- and novelty-seeking, in the sense that this behavioral strategy may disambiguate context and hence reduce uncertainty (Bromberg-Martin and Monosov, 2020; Monosov, 2024). Although originally proposed to account for extinction learning, where the CS becomes ambiguous early in extinction, this interpretation can be extended to autoshaping under uncertain reward conditions. RPE signals may trigger attentional shifts and exploratory behavior aimed at reducing uncertainty. For example, a dog that repeatedly retrieves a thrown object focuses exclusively on the task; but if the owner pretends to throw and hides the object instead, the dog quickly shifts to sniffing and searching the surroundings. This attentional reorientation and exploration occur because the previously rewarded CS (the throwing gesture) fails to predict the expected outcome. Similar patterns are observed in operant settings, where early extinction produces heightened behavioral vigor (Shahan, 2022) and increased behavioral variability (Donoso et al., 2021). A comparable process may underlie sign-tracking amplification when a cue fails to fully predict reward, an effect that likely reflects adaptive search behavior rather than a mere failure of learning or a purely appetitive reaction. This functional view of RPE is consistent with our suggestion that behavioral invigoration under reward uncertainty reflects an adaptive response to a challenge that occurs in the form of CS ambiguity (see also Anselme, 2025).

Dopamine release during uncertainty-related behaviors helps explain why gambling, and gambling-like situations in animals, can become addictive. Evidence shows that uncertainty amplifies or accelerates the sensitization process central to the incentive sensitization theory of addiction. For example, Gottlieb (2006) reported that pigeons trained under uncertain reward conditions pecked a cue far more than those trained with predictable rewards, and this heightened response persisted even when the cue was replaced by a novel stimulus. Similarly, Robinson M. J. F. et al. (2014) exposed rats to sequences of high, moderate, or low uncertainty in autoshaping tasks. Rats that started with high uncertainty continued to respond more vigorously to cues later, regardless of recent conditions. Converging findings across species suggest that early exposure to uncertainty can produce lasting behavioral sensitization, effects resembling those seen with drugs of abuse and in gambling-like tasks (Mascia et al., 2019; Robinson et al., 2015; Singer et al., 2012; Swintosky et al., 2021; Zack et al., 2014).

Dopamine release under uncertainty might also offer clues about addiction. It is possible that addictive drugs, by artificially triggering phasic dopamine surges, mimic the motivational amplification observed under unpredictable reward conditions. If so, these drugs could accelerate incentive sensitization and create an exaggerated motivational state, somewhat analogous to the invigoration produced by uncertainty, but without the natural constraints that typically limit this effect in everyday environments. This interpretation closely follows Redish's (2004) computational account of addiction in which addictive drugs introduce a non-compensable dopaminergic prediction error that prevents the value function from converging. Because this error cannot be canceled through learning, the value of drug-associated states increases progressively, biasing action selection toward drug-seeking despite stable or even superior alternative rewards. Reward uncertainty may constitute a functional analog of this process. When CS–US contingencies are probabilistic, the prediction error signal cannot be fully driven to zero, allowing dopaminergic responses to persist across trials and sustaining value updating. However, a motivational account is necessary to explain the behavioral effects of this process and, in an addictive context, how this process can persist and cause relapse, even after months or years of abstinence (Robinson and Berridge, 2025).

Recent developments in Redish's framework provide further support for the idea that uncertainty can produce addiction-like motivational amplification. While the original model (Redish, 2004) attributed addictive behavior to a non-compensable dopaminergic prediction error induced by drug pharmacology, later work by his group has generalized this mechanism to broader failures of internal model updating. Kalhan et al. (2021) show that dysfunctions in Anterior Cingulate Cortex dopamine circuits lead to misaligned internal representations in which cues with high apparent salience—such as drug-predictive stimuli—are overweighted during learning, while other cues and negative feedback are underweighted. More recently, Kalhan et al. (2023) formalized these ideas in a computational salience weighting model, demonstrating that any condition producing elevated or volatile prediction errors (including environmental uncertainty) can produce asymmetric learning, reduced sensitivity to costs, steeper delay discounting, and persistent cue-driven behavior. This suggests that reward uncertainty, by maintaining high prediction error signals, may drive the same kind of salience misattribution and value distortion attributed to addictive drugs in the initial Redish model. Interpretations in line with this account have been recently proposed by Zack et al. (2020), who suggest that gambling disorder might be a “*sensitization-like syndrome caused in part by chronic exposure to intermittent, unpredictable reward and mediated by sustained hyper-reactivity of brain dopamine pathways*” (p. 5). The same computational logic extends to modern digital environments. As recently noted (Clark and Zack, 2023; White et al., 2024), features such as infinite scrolling, unpredictable notifications, and swipe-to-refresh interfaces replicate variable-ratio reward schedules, maximizing anticipation and uncertainty. These design features might maintain elevated prediction errors, bias learning toward positive outcomes, and reduce the impact of negative feedback. Consistent with this view, design features of online applications that rely on variable reinforcement schedules have been suggested to promote dysregulated and addictive online behaviors (Flayelle et al., 2023).

### 2.4 Conclusion

Evidence from animal studies demonstrates that reward-predictive cues can acquire strong motivational and attentional properties through Pavlovian learning, driving approach behavior even in the absence of instrumental contingencies. Critically, when reward delivery is uncertain, these cue-triggered responses are markedly amplified: sign-tracking intensifies, dopaminergic activity surges, and attention becomes disproportionately biased toward reward-associated cues. This pattern suggests that uncertainty acts as a powerful enhancer of persistent and sometimes maladaptive engagement with predictive stimuli. These processes operate largely automatically, independent of explicit expectations or goal-directed control. Addictive drugs might mimic these uncertainty-driven effects by artificially inducing phasic dopamine release, thereby accelerating incentive sensitization and promoting compulsive cue attraction. While this hypothesis remains to be fully demonstrated, it offers a compelling framework for understanding parallels between uncertainty and drug-induced motivational effects. Taken together, the evidence suggests that uncertainty functions as a potent modulator of cue-driven motivation, providing a mechanistic bridge between adaptive learning strategies and the compulsive behaviors characteristic of addiction.

## 3 Human research

### 3.1 Evidence for incentive motivation and sing-tracking behaviors

Following the same logic as in the animal research section, we first review human research on motivation and attention (Sections 3.1 and 3.2) before discussing the relatively limited number of studies explicitly examining reward uncertainty in humans (Section 3.3). Research on incentive motivation and sign-tracking has been extensively conducted in laboratory animals since the late twentieth century (Boakes, 1977; Hearst and Jenkins, 1974). However, systematic empirical investigations of these phenomena in humans have only begun to emerge more recently (Anselme and Robinson, 2020; Colaizzi et al., 2020; Heck et al., 2025a). In a recent scoping review, we showed that the paradigms used to assess human sign-tracking are considerably more diverse than those employed in animal studies, and that no consensus yet exists regarding the most appropriate methods to identify individual differences in sign- and goal-tracking behaviors (Heck et al., 2025a). Among the available procedures, Pavlovian conditioned approach (PCA) tasks remain the closest human analog to the traditional autoshaping paradigm used in animals, and they have been instrumental in demonstrating the expression of incentive salience in humans. PCA tasks can be implemented in a physical format, allowing direct interaction with a predictive cue and a reward receptacle, as in animal research, or in a virtual, computerized format, where eye-movement measures are typically used to infer sign- vs. goal-tracking tendencies. Across formats, PCA tasks rely on an initially neutral cue (CS) acquiring predictive value through its association with a reward (US). Whereas food rewards are universally used in animal autoshaping, human studies more commonly employ monetary incentives, often represented by images of coins or banknotes (Cherkasova et al., 2024; Duckworth et al., 2022; Garofalo and di Pellegrino, 2015; Schad et al., 2020; Schettino et al., 2024). Figure 1B illustrates sign- and goal-tracking behaviors in a human PCA task.

Recent findings provide preliminary support for the existence of ST and GT phenotypes in humans, both in physical implementations of PCA tasks (Colaizzi et al., 2023; Cope et al., 2023) and in computerized versions (Garofalo and di Pellegrino, 2015; Heck et al., 2025b; Schad et al., 2020; Schettino et al., 2024). However, identifying clear-cut phenotypes in humans is considerably more challenging than in animal models, often resulting in intermediate or ambiguous categories such as “non-ST” or “non-GT” (e.g., Colaizzi et al., 2023; Heck et al., 2025b; Joyner, 2019). Several human PCA studies (e.g., Garofalo and di Pellegrino, 2015; Lehner et al., 2017; Schad et al., 2020) have incorporated a non-reinforced cue (CS–) in addition to the reinforced CS+. The two cues are perceptually identical, making the CS– a useful control for determining whether preferential attention to the CS+ reflects genuine motivational attraction rather than a non-specific attentional bias. Consistent with findings from animal autoshaping, robust evidence shows that some participants attend to the CS+ more than to the US location, supporting the involvement of incentive salience in these tasks. Paralleling animal studies, several human investigations have also reported associations between sign-tracking tendencies and measures of impulsivity (Colaizzi et al., 2023; Cope et al., 2023; Garofalo and di Pellegrino, 2015; Heck et al., 2025b). Nonetheless, substantial heterogeneity in experimental methods and analytic approaches, along with the presence of non-significant findings, often found in dissertations rather than published articles (Colom, 2023; Doran, 2016; Joyner, 2019), raises concerns about potential publication bias and limits the strength of current conclusions, although some interesting non-significant findings have also been published (Cherkasova et al., 2024; Dinu et al., 2024). More broadly, research using human PCA paradigms still lacks sufficient replication and validation, making it premature to assert that stable ST and GT phenotypes exist in humans (Heck et al., 2025a). Addressing this issue, a recent study by Badioli et al. (2026) examined the test–retest reliability of an eye-gaze-based index over a 4-month interval. Although the measure demonstrated good reliability for detecting sign-tracking, it showed poor consistency for identifying goal-tracking, yielding suboptimal overall reliability. These findings underscore the need for further methodological refinement and the development of more robust and standardized measures before stable phenotypic classifications can be confidently established in human populations.

In conclusion, most human PCA studies have focused primarily on the motivational and behavioral aspects of sign- and goal-tracking, while giving comparatively little attention to related processes such as attentional prioritization of reward-related cues or the influence of reward uncertainty on attentional and behavioral performance. Yet, as recently emphasized by Le Pelley et al. (2024), cues that acquire incentive salience are especially likely to capture attention, thereby increasing their impact on subsequent behavior. These authors further argue that a comprehensive understanding of motivated behavior requires acknowledging how selective attention shapes the encoding and evaluation of stimuli, processes that ultimately guide actions and decisions.

### 3.2 Focus on motivated attention: value-modulated attentional capture studies

Although incentive salience and sign-tracking behaviors have only been modestly explored in humans using PCA paradigms, a substantial literature has examined how reward history shapes attentional prioritization, leading to closely related conclusions. Attention can be defined as “*the cognitive mechanisms used to allocate mental resources to the processing of certain aspects of sensory inputs*” (Le Pelley et al., 2015; p. 4). But why do we preferentially attend to some stimuli rather than others? A widely accepted framework distinguishes three processes that bias attention toward particular stimuli (Theeuwes, 2019). The first is stimulus-driven bottom-up attention, whereby physically salient stimuli (e.g., those that are bright, large, or loud) automatically capture attention. The second is goal-directed top-down attention, which is strategically deployed toward stimuli relevant to an individual's current objectives. The third is “selection history,” reflecting persistent attentional biases toward stimuli that have been associated with reward or other significant outcomes in the past, independent of physical salience or current goals. This last category is especially relevant for motivated attention: for instance, one may instantly notice a €2 coin on the ground not because it is perceptually striking, but because it is motivationally salient due to its learned value. Indeed, a considerable body of research shows that rewards can act as a potent source of incentive motivation for the control of attention (Chelazzi et al., 2013; Failing and Theeuwes, 2018), enhancing performance across a variety of tasks by increasing the priority of reward-associated stimuli.

The value-modulated attentional capture (VMAC) paradigm was specifically designed to examine how reward history shapes motivated attention. Whereas PCA paradigms focus on overt approach behavior, VMAC paradigms quantify the attentional consequences of incentive salience by measuring how strongly reward-predictive cues draw gaze or slow down task performance. VMAC refers to the robust finding that stimuli previously associated with higher rewards capture attention more effectively than those linked to lower rewards, even when such capture is detrimental to the task goal (Le Pelley et al., 2015, 2019). In the standard VMAC task, each trial begins with a central fixation cross, followed by a visual search display composed of six shapes, five circles and one diamond, arranged symmetrically around the cross (Figure 1C). The diamond target and four of the circles appear in gray, whereas the remaining circle act as a distractor and is colored according to the reward magnitude it predicts (e.g., green for a high-value cue worth 500 points, orange for a low-value cue worth 10 points). Participants are instructed to identify the orientation of a line segment within the diamond (vertical or horizontal). Points are awarded based on response speed and accuracy, and cumulative performance determines the final amount of money/points obtained. A commonly used variant replaces manual responses with eye-movements, requiring participants to locate the diamond as quickly as possible. Even though ignoring the distractors is the optimal strategy, participants reliably exhibit attentional capture by the high-value distractors. Eye-tracking studies show that gaze is disproportionately drawn toward these colored stimuli, while reaction-time data reveal slower detection of the target when high-value distractors are present (Le Pelley et al., 2015; Pearson et al., 2015). Such findings indicate that reward-associated cues acquire an elevated attentional priority, consistent with the idea that motivationally salient stimuli can exert automatic, bottom-up influence on attentional allocation, even when this conflicts with top-down goals.

The VMAC effect has been proposed as a potential human analog of the sign-tracking behavior observed in animal autoshaping (Albertella et al., 2019; Byrom and Murphy, 2018; Le Pelley et al., 2015, 2024; Wiers et al., 2021). This parallel is based on their shared behavioral bases: in both cases, a stimulus predictive of reward acquires incentive salience, thereby biasing attention and/or approach behavior toward it. In animal autoshaping, sign-tracking is expressed through overt approach and physical interaction with the reward-predictive cue (e.g., a lever). In contrast, in human VMAC paradigms, sign-tracking is inferred indirectly from attentional capture, manifested as slower identification of the target when a high-value distractor is present. Although the behavioral readouts differ (approach vs. attentional interference), the underlying mechanism is conceptually similar: reward-associated cues acquire motivational significance that competes with, and at times overrides, goal-directed control. Whether in autoshaping or VMAC tasks, attention and motivation are tightly intertwined. In both cases, cues endowed with incentive salience exert a disproportionate influence on behavior, revealing a common mechanism through which reward history shapes perceptual prioritization and action. Figure 1 provides a visual overview of sign-tracking and goal-tracking behaviors, as observed in PCA tasks in both nonhuman and human animals, as well as in VMAC tasks.

Yet, the VMAC paradigm does not fully reproduce the defining characteristics of animal autoshaping procedures (Anselme and Robinson, 2020; Colaizzi et al., 2020; Heck et al., 2025a). Several key differences are notable. First, reward delivery in VMAC is not response-independent; participants must respond correctly and within a specific time window to obtain points. Second, the paradigm does not allow a direct comparison between cue-directed and reward-directed approach behaviors, a central feature of autoshaping used to distinguish sign- and goal-tracking profiles. Third, VMAC tasks typically include instrumental contingencies, such as reward omission following incorrect or delayed responses, that are absent from classical Pavlovian procedures in animals. Despite these structural differences, there is no evidence that VMAC fails to capture processes related to incentive salience. Rather, it measures these processes indirectly, through the degree of attentional interference produced by reward-associated distractors. Importantly, findings from VMAC studies converge remarkably well with decades of animal research, particularly in their consistent associations with addiction-related traits and behaviors (e.g., Albertella et al., 2017, 2019, 2021; Liu et al., 2021; Watson et al., 2024). Consequently, although VMAC may be less suited than PCA paradigms for identifying clear sign- and goal-tracking phenotypes in humans, it nevertheless provides a powerful tool for capturing differential cue attraction and attentional bias for reward cues. Consistent with this, a recent study achieved the first successful back-translation of the VMAC paradigm to mice and rats (Bradfield et al., 2026), showing that reward-predictive distractors impair instrumental performance in rodents in a manner closely mirroring VMAC effects in humans. Notably, the VMAC effect persisted following outcome devaluation via conditioned taste aversion, indicating that the attentional disruption cannot be explained by simple sign-tracking alone or habitual responding, but instead points to a more compulsive process. However, these conclusions are based on relatively small samples, and while the approach is elegant and the findings promising, replication across laboratories and conditions will be essential to establish rodent VMAC as a robust back-translational model of cue-driven attentional capture.

VMAC effects were originally interpreted within the selection-history framework, according to which prior stimulus–reward contingencies leave a persistent trace that biases attention toward previously rewarded cues, even when such attentional capture is detrimental to current task performance (Le Pelley et al., 2015). At a functional level, it has been suggested that reward selection history changes a stimulus' representation, rendering it more salient to the visual system (Failing and Theeuwes, 2018; Theeuwes, 2019). As Failing and Theeuwes (2018) noted, this mechanism closely mirrors incentive salience: a previously neutral cue is reshaped at the neural level so that, through its link to a rewarding outcome, it becomes a powerful attractor of attention. Thus, a complementary interpretation views VMAC through a motivational lens, proposing that reward-associated cues attract attention because they elicit a conditioned “approach-like” response mediated by dopaminergic signaling. From this perspective, the learned association between a stimulus and its reward outcome enhances its incentive salience, thereby granting it attentional priority even when this conflicts with the individual's explicit goals. Supporting this view, converging neurobiological evidence shows that striatal dopaminergic mechanisms contribute directly to VMAC. Anderson et al. (2014) demonstrated that the striatum, a central structure of the basal ganglia involved in motivational processing, generates an attentional priority signal for reward-predictive stimuli. Extending these findings, a subsequent PET study revealed that dopamine release in the dorsal striatum predicts the magnitude of VMAC, with higher dopaminergic activity corresponding to stronger attentional capture by high-value distractors (Anderson et al., 2016). These results are particularly noteworthy given the well-established role of dopamine in attributing incentive salience to reward cues (Berridge and Robinson, 1998) and in the broader neurobiology of addiction (Robinson and Berridge, 1993, 2025). Together, they suggest that the attentional biases observed in VMAC reflect the same dopaminergic processes that underlie cue-triggered motivation in animal models, reinforcing the translational link between motivational attention and addiction-related behaviors.

The role of dopamine in motivated attention has also been examined through direct comparisons of human sign-trackers and goal-trackers. In a study by Schad et al. (2020), sign-tracking was operationalized using a Pavlovian-to-instrumental transfer (PIT) task, in which a Pavlovian cue–reward association facilitates subsequent instrumental responding. Eye-tracking, pupillometry, fMRI and computational modeling were used. The results indicated that human sign-tracking is primarily driven by model-free, dopamine-dependent processes that endow cues with motivational value and influence both attention and physiological markers such as gaze patterns and pupil dilation. In contrast, goal-tracking behavior appeared to rely more heavily on model-based computations supported by higher-order cognitive processes. This dissociation closely mirrors findings in animal research: sign-trackers exhibit heightened mesolimbic dopamine activity but reduced cortical acetylcholine modulation, resulting in poorer sustained attention and stronger cue attraction, whereas goal-trackers show enhanced cholinergic regulation and more stable attentional performance (Flagel et al., 2011a; Paolone et al., 2013; Sarter and Phillips, 2018). Consistent with this framework, Albertella et al. (2017) reported that VMAC was associated with illicit drug use only among individuals with low top-down control of selective attention, suggesting that weaker top-down regulation increases vulnerability to cue-driven, dopamine-mediated behavior. Together, these findings reinforce the view that human individual differences in motivated attention reflect underlying distinctions analogous to those observed in animal models.

### 3.3 Effects of uncertainty on human motivated attention

Although the impact of uncertainty on attentional and motivational processes, especially on sign-tracking, has been well documented in nonhuman animals, systematic investigation of these mechanisms in humans is relatively recent. This emerging line of research has largely relied on VMAC-based procedures in which the reward associated with a distractor is made inconsistent, giving rise to what has been termed uncertainty-modulated attentional capture (UMAC). The UMAC paradigm offers a controlled approach to examine how unpredictable reward outcomes alter the attentional priority of cues, thereby providing a human analog to uncertainty-driven effects observed in animal conditioning studies.

The UMAC paradigm introduced by Le Pelley et al. (2019) closely mirrors the traditional VMAC design, with one critical modification: one distractor is associated with either a high reward (e.g., 500 points) or a low reward (e.g., 10 points) with equal probability (50/50: called the “non-predictive” distractor by the authors, since the upcoming reward magnitude cannot be anticipated). This manipulation introduces explicit, high reward uncertainty (50%) while keeping physical stimulus properties constant, allowing researchers to isolate the effects of unpredictability on attentional capture.

A growing number of studies have now shown that inconsistent distractors, i.e., stimuli associated with unpredictable reward outcomes, capture attention more strongly than fully predictive distractors (Cho and Cho, 2021; Chow et al., 2025; Ju and Cho, 2023; Le Pelley et al., 2019; Massa et al., 2024; Pearson et al., 2024). These findings align with the uncertainty principle proposed by Pearce and Hall (1980), which posits that cues with a high uncertainty attract attention more because their potential consequences require increased monitoring. They also parallel evidence from animal research showing that uncertain cues elicit heightened motivational and attentional engagement, reflected in enhanced sign-tracking (Anselme et al., 2013; Glueck et al., 2018; Kaneko et al., 2025). Classical learning theories have long debated whether attentional allocation is better explained by the uncertainty principle (Pearce and Hall, 1980) or by the predictiveness principle (suggesting, conversely, that more reliable cues attract greater attention, Mackintosh, 1975), for which evidence has accumulated (e.g., Degni et al., 2025; Trick et al., 2011). Although Le Pelley et al.'s (2016) review concluded that predictiveness effects were historically more robust in human studies, the authors noted that many earlier paradigms may not have been suited to adequately test the role of uncertainty. More recent work has renewed both theoretical and empirical support for uncertainty-driven attentional prioritization in humans (Beesley et al., 2015; Folk and Anderson, 2010; Frings et al., 2019; Morriss et al., 2024). Together, these findings indicate that, under appropriate task conditions, uncertainty can serve as a powerful driver of attentional priority, mirroring mechanisms documented in nonhuman species and reinforcing the translational relevance of uncertainty-modulated motivated attention.

Additional evidence suggests that the influence of uncertainty on attention is both rapid and largely automatic. For instance, UMAC persists even when participants are explicitly informed that the inconsistent distractor is non-predictive, showing that the effect cannot be reduced to conscious expectations (Chow et al., 2025). In line with this, Le Pelley et al. (2019) reported that attentional capture by inconsistent distractors is maximal on the first eye saccade, demonstrating an immediate, stimulus-driven bias (see also Ju and Cho, 2023). Cho and Cho (2021) further observed that this effect is transient under some conditions, emerging during the initial block of trials but diminishing thereafter.

These results suggest that UMAC may be short-lived depending on the experimental context. However, several studies have documented sustained uncertainty effects across multiple blocks (Le Pelley et al., 2019; Massa et al., 2024), indicating that the persistence of UMAC varies as a function of task parameters or of the strength of the learned reward associations. Animal research reveals a contrasting pattern. Once uncertainty invigorates sign-tracking, the effect tends to be robust and enduring across repeated trials and sessions (e.g., Robinson M. J. F. et al., 2014). These species differences are not necessarily incompatible. In humans, rapid habituation of the automatic orienting response may reflect a diminishing need to monitor an inconsistent cue. In contrast, in animals, the motivational arousal triggered by uncertainty remains elevated as long as unpredictability persists. This disparity may be especially evident in physical PCA procedures, such as autoshaping, where behavior provides a more direct expression of incentive motivation than eye-movement-based measures do. It may also reflect the fact that sign-tracking in animals is less constrained by top-down attentional control. Moreover, human studies indicate that multiple forms of uncertainty contribute to UMAC (Cho and Cho, 2021; Ju and Cho, 2023; Pearson et al., 2024), paralleling the effects of probabilistic and temporal uncertainty observed in rodent autoshaping paradigms (Anselme et al., 2013; Kaneko et al., 2025).

Taken together, these findings suggest that uncertainty modulates motivated attention through mechanisms that are broadly conserved across species. Preliminary evidence also supports a translational account in which reward uncertainty drives dopamine release, an effect well established in animal studies but not yet directly examined in the context of UMAC. Although no human study has empirically tested the link between dopaminergic activity and uncertainty-modulated attentional capture, the animal literature showing that uncertainty enhances dopaminergic signaling (Fiorillo et al., 2003; Hart et al., 2015; Schultz, 1998) provides a strong rationale for anticipating a similar relationship in humans.

### 3.4 Conclusion

Human research converges with animal findings in showing that reward-associated cues exert a powerful influence on attention and motivation, often overriding goal-directed intentions. Under conditions of reward uncertainty, these effects are further amplified: cues associated with variable or unpredictable rewards attract disproportionate attention and elicit stronger approach-related tendencies. This uncertainty-modulated attentional capture reflects rapid and largely automatic processes, likely supported by dopaminergic mechanisms, that bias behavior toward reward-predictive stimuli regardless of explicit knowledge or conscious strategies. Such cue reactivity reveals an implicit motivational pull that persists even when individuals attempt to ignore or suppress it. Overall, the human literature suggests that uncertainty enhances both the attentional and motivational effects of reward cues, possibly reinforcing the translational link between uncertainty-driven cue engagement and the mechanisms underlying addictive behaviors.

## 4 The adaptive function of cue-triggered behavior

The association between sign-tracking and vulnerability to addiction should not obscure the adaptive origins of cue-triggered behavior. In natural environments, predictive cues and rewards are rarely separable: the perceptual features of prey, food odors, or mate-related signals typically provide reliable, proximal access to biologically significant outcomes. In many contexts, particularly when rewards are distal or partially hidden, using the cue itself as a motivational signal is advantageous. This functional role helps explain why sign-tracking is evolutionarily conserved across diverse species. As noted by Krause and Domjan (2017), Pavlovian conditioning evolved because biologically important events are systematically preceded by early, predictive cues embedded within natural environments. A parallel perspective was suggested in the human attentional selection literature, where the tendency for reward-related stimuli to capture attention has likewise been framed as an adaptive (evolutionary shaped) process (Della Libera and Chelazzi, 2006, 2009; Pitchford and Arnell, 2025).

Uncertainty often enhances the adaptive value of cue responding. Inconsistent or ambiguous cues draw attention (Esber and Haselgrove, 2011; Pearce and Hall, 1980) because they signal that a greater engagement in the task is required (but see Mackintosh, 1975; O'Reilly et al., 2013; Wittmann et al., 2016) Under such conditions, sign-tracking not only intensifies but also becomes more prevalent across individuals (Robinson et al., 2015). Amplification effects observed in animals, such as prolonged engagement with cues, greater response vigor, and increased behavioral variability (e.g., Blaisdell et al., 2016; Fuentes-Verdugo et al., 2020), can be understood functionally as strategies that increase reward discovery in sparse or unpredictable environments (Anselme and Güntürkün, 2018). Computational foraging simulations support this view. Organisms exposed to fewer, less predictable cues show greater exploratory variability and improved survival (Anselme et al., 2017, 2018).

In humans, parallels between UMAC and uncertainty-driven attentional exploration have been discussed (Cho and Cho, 2021; Chow et al., 2025; Ju and Cho, 2023; Le Pelley et al., 2019; Pearson et al., 2024). These findings align with exploration–exploitation accounts of decision making, which posit that uncertainty promotes exploratory attention to reduce future uncertainty (Beesley et al., 2015; Gottlieb et al., 2013). From this perspective, uncertainty-related attentional capture reflects a functional mechanism designed to update internal models of reward contingencies rather than maladaptive distraction.

However, human studies have not yet demonstrated the full range of “amplification” effects observed in animals, such as increased cue-directed vigor or behavioral variability. These phenomena extend beyond attentional capture and require a motivational framework capable of explaining how uncertainty energizes behavior and why repeated exposure to inconsistent cues can foster addiction-like patterns, particularly in gambling contexts (Anselme, 2025; Anselme and Güntürkün, 2019). A comprehensive theory should therefore integrate both the functional, evolutionarily conserved aspects of sign-tracking and its maladaptive potential in modern environments where reward cues are artificially intensified and uncertainty is systematically engineered.

## 5 General discussion

Across this review, we examined how reward-predictive cues shape motivated behavior and attentional allocation in both humans and nonhuman animals, and how these processes are strongly modulated by reward uncertainty. Animal evidence shows that uncertain or partially reinforced cues elicit heightened sign-tracking behaviors, increased dopaminergic responses, and persistent approach tendencies (Anselme et al., 2013; Hellberg et al., 2019; Mascia et al., 2019; Robinson M. J. F. et al., 2014). Parallel findings in humans, particularly from UMAC paradigms, demonstrate that inconsistent reward cues attract greater attention than fully predictive ones, even when this impairs task performance (Cho and Cho, 2021; Chow et al., 2025; Ju and Cho, 2023; Pearson et al., 2024). Together, these observations indicate that uncertainty amplifies cue-triggered motivation and attentional capture through rapid, automatic mechanisms operating largely outside conscious control.

The convergence of human and animal data suggests that sign-tracking under uncertainty and UMAC rely on shared mechanisms, likely involving dopaminergic systems that attribute incentive salience to ambiguous cues (Hart et al., 2015; Ju and Cho, 2023; Mascia et al., 2019; Massa et al., 2024; Robinson et al., 2015; Robinson and Anselme, 2019). Functionally, these mechanisms may reflect evolutionarily conserved strategies that promote exploration and information-seeking in unpredictable environments (Anselme, 2025; Bromberg-Martin and Monosov, 2020; Frings et al., 2019; Pearson et al., 2024). Heightened attention to uncertain cues is therefore not inherently maladaptive; rather, it can support the updating of internal models of reward contingencies.

A longstanding debate in associative learning concerns whether organisms allocate attention to cues because they reliably predict important outcomes or because they remain uncertain and therefore informative. According to the predictiveness principle (Mackintosh, 1975), attention is preferentially directed toward cues that most accurately signal outcomes, facilitating exploitation of stable contingencies. Conversely, the uncertainty principle (Pearce and Hall, 1980) proposes that attention is allocated to ambiguous cues to promote exploratory learning. Contemporary accounts converge on the idea that both mechanisms operate in parallel, with their influence varying according to task demands and learning history.

Human studies support this dual-process view. For example, Easdale et al. (2019) showed that cues with high predictive validity receive greater attention, whereas unexpected uncertainty enhances associability and accelerates learning of new contingencies. This reciprocal relationship highlights how learned predictive value guides attention, while attention modulates the rate of associative learning.

However, greater attention to uncertain cues does not imply that all cue-triggered behaviors follow an uncertainty-driven pattern. Studies using Pavlovian-to-instrumental transfer (PIT) tasks point to a dissociation between attentional orienting and motivational influence. Trick et al. (2011) found that dwell time peaked for cues of intermediate predictiveness, whereas PIT effects increased with cue predictiveness. Therefore, uncertainty promotes attentional exploration, whereas motivational invigoration of instrumental responding follows a predictiveness gradient. Recent findings further support this distinction. Degni et al. (2025) demonstrated that weakening cue-outcome contingencies during Pavlovian training reduces the subsequent bias exerted by Pavlovian cues on instrumental decisions, suggesting that cue predictiveness, not uncertainty, drives maladaptive Pavlovian interference in value-based choice.

These observations carry important implications for addiction and gambling-related behaviors. From a Mackintosh perspective, drug-associated cues that consistently predict pharmacological reward acquire strong motivational control, consistent with cue-triggered craving and relapse. In contrast, the Pearce–Hall framework explains why uncertain or intermittently reinforced cues, common in gambling environments, remain highly attention-capturing despite limited predictive value. Addiction may reflect a pathological decoupling of these systems: uncertain cues dominate attentional orienting and learning, whereas highly predictive cues exert disproportionate motivational control over action. Addictive substances may further exacerbate this imbalance by generating persistent prediction-error-like signals (Redish, 2004), thereby maintaining both heightened cue salience and strong motivational impact.

From a neurobiological perspective, addictive drugs may artificially mimic and amplify the motivational effects normally produced by uncertainty. Pharmacologically induced dopaminergic surges can endow drug-associated cues with exaggerated incentive salience, bypassing natural constraints on uncertainty-driven learning. In this sense, addiction may hijack an evolutionarily adaptive system by generating an artificial form of uncertainty-like stimulation, rendering drug cues irresistibly attractive and persistently attention-capturing.

Despite these conceptual parallels, important translational challenges remain. Although the neural bases of uncertainty-driven cue attraction have been characterized in animals (Fiorillo et al., 2003; Hart et al., 2015), the relevant biological mechanisms in humans are only beginning to be investigated. Dopaminergic involvement has been demonstrated in value-modulated attentional capture (Anderson et al., 2014, 2016), but direct evidence linking dopamine release to UMAC is still lacking, and the role of stress-related systems such as the HPA axis has yet to be systematically assessed. Moreover, key behavioral amplifications observed in animals under uncertainty, such as heightened vigor, persistence, or increased variability, have not yet been systematically explored in humans.

Strengthening the translational bridge between species will require several methodological advances. Future research should (1) develop standardized paradigms for assessing cue-driven behaviors in humans, including analogs of sign-tracking; (2) systematically manipulate distinct dimensions of uncertainty (probability, magnitude, timing) to more closely match animal studies; and (3) investigate the neurobiological substrates of UMAC, with particular attention to dopaminergic and HPA-axis mechanisms. Additionally, identifying individual differences in susceptibility to uncertainty-modulated attention will be essential for determining who is most vulnerable to maladaptive cue reactivity.

Clarifying how uncertainty shapes motivated attention across species has significant implications for both basic neuroscience and psychopathology. Mechanisms that evolved to support adaptive exploration in uncertain environments can, in modern contexts such as gambling or drug use, be co-opted or exaggerated by artificial dopaminergic stimulation. A deeper understanding of these processes will refine theoretical models of incentive salience and attentional prioritization, while informing prevention and intervention strategies targeting maladaptive cue reactivity, whether driven by uncertainty, by addictive substances, or by their interaction.

## References

[B1] Ahrens A. M. Singer B. F. Fitzpatrick C. J. Morrow J. D. Robinson T. E. (2016). Rats that sign-track are resistant to Pavlovian but not instrumental extinction. Behav. Brain Res. 296, 418–430. doi: 10.1016/j.bbr.2015.07.055 26235331 PMC4681397

[B2] Albertella L. Copeland J. Pearson D. Watson P. Wiers R. W. Le Pelley M. E. (2017). Selective attention moderates the relationship between attentional capture by signals of nondrug reward and illicit drug use. Drug Alcohol Depend. 175, 99–105. doi: 10.1016/j.drugalcdep.2017.01.041 28411561

[B3] Albertella L. Vd Hooven J. Bovens R. Wiers R. W. (2021). Reward-related attentional capture predicts non-abstinence during a one-month abstinence challenge. Addict. Behav. 114:106745. doi: 10.1016/j.addbeh.2020.106745 33310691

[B4] Albertella L. Watson P. Yücel M. Le Pelley M. E. (2019). Persistence of value-modulated attentional capture is associated with risky alcohol use. Addict. Behav. Rep. 10:100195. doi: 10.1016/j.abrep.2019.100195 31245528 PMC6582188

[B5] Anderson B. A. Kuwabara H. Wong D. F. Gean E. G. Rahmim A. Brašić J. R. . (2016). The role of dopamine in value-based attentional orienting. Curr. Biol. 26, 550–555. doi: 10.1016/j.cub.2015.12.062 26877079 PMC4767677

[B6] Anderson B. A. Laurent P. A. Yantis S. (2014). Value-driven attentional priority signals in human basal ganglia and visual cortex. Brain Res. 1587, 88–96. doi: 10.1016/j.brainres.2014.08.062 25171805 PMC4253668

[B7] Anselme P. (2016). Motivational control of sign-tracking behaviour : a theoretical framework. Neurosci. Biobehav. Rev. 65, 1–20. doi: 10.1016/j.neubiorev.2016.03.014 27016362

[B8] Anselme P. (2025). Unconscious will as a neurobehavioral mechanism against adversity. Neurosci. Biobehav. Rev. 169:105985. doi: 10.1016/j.neubiorev.2024.105985 39709153

[B9] Anselme P. Güntürkün O. (2018). How foraging works : uncertainty magnifies food-seeking motivation. Behav. Brain Sci. 42:e35. doi: 10.1017/S0140525X18000948 29514723

[B10] Anselme P. Güntürkün O. (2019). Incentive hope : a default psychological response to multiple forms of uncertainty. Behav. Brain Sci. 42:e58. doi: 10.1017/S0140525X18002194 30940273

[B11] Anselme P. Otto T. Güntürkün O. (2017). How unpredictable access to food increases the body fat of small passerines : a mechanistic approach. Behav. Processes 144, 33–45. doi: 10.1016/j.beproc.2017.08.013 28870644

[B12] Anselme P. Otto T. Güntürkün O. (2018). Foraging motivation favors the occurrence of Lévy walks. Behav. Processes 147, 48–60. doi: 10.1016/j.beproc.2017.12.014 29274764

[B13] Anselme P. Robinson M. J. F. (2020). From sign-tracking to attentional bias : implications for gambling and substance use disorders. Prog. Neuropsychopharmacol. Biol. Psychiatry 99:109861. doi: 10.1016/j.pnpbp.2020.109861 31931091

[B14] Anselme P. Robinson M. J. F. Berridge K. C. (2013). Reward uncertainty enhances incentive salience attribution as sign-tracking. Behav. Brain Res. 238, 53–61. doi: 10.1016/j.bbr.2012.10.006 23078951 PMC4066390

[B15] Badioli M. Danti C. Degni L. Finotti G. Bernardi V. Mattioni L. . (2026). Test-retest reliability of the gaze index for sign-tracking and goal-tracking. Behav. Res. Methods 58:44. doi: 10.3758/s13428-025-02919-5 41559434

[B16] Barrus M. M. Winstanley C. A. (2016). Dopamine D3 receptors modulate the ability of win-paired cues to increase risky choice in a rat gambling task. J. Neurosci. 36, 785–794. doi: 10.1523/JNEUROSCI.2225-15.2016 26791209 PMC6602008

[B17] Beesley T. Nguyen K. P. Pearson D. Le Pelley M. E. (2015). Uncertainty and predictiveness determine attention to cues during human associative learning. Q. J. Exp. Psychol. 68, 2175–2199. doi: 10.1080/17470218.2015.1009919 25832459

[B18] Berridge K. (2003). “Irrational pursuits: hyper-incentives from a visceral brain,” in The Psychology of Economic Decisions, Vol. 1, eds. I. Brocas, and J. D. Carrillo (Oxford: Oxford University Press),17–40. doi: 10.1093/oso/9780199251063.003.0002

[B19] Berridge K. C. (2023). Separating desire from prediction of outcome value. Trends Cogn. Sci. 27, 932–946. doi: 10.1016/j.tics.2023.07.007 37543439 PMC10527990

[B20] Berridge K. C. Robinson T. E. (1998). What is the role of dopamine in reward : hedonic impact, reward learning, or incentive salience? Brain Res. Rev. 28, 309–369. doi: 10.1016/S0165-0173(98)00019-8 9858756

[B21] Biback C. Zack M. (2015). The relationship between stress and motivation in pathological gambling: a focused review and analysis. Curr. Addict. Rep. 2, 230–239. doi: 10.1007/s40429-015-0064-9

[B22] Bindra D. (1978). How adaptive behavior is produced: a perceptual-motivational alternative to response-reinforcement. Behav. Brain Sci. 1, 41–91. doi: 10.1017/S0140525X00059380

[B23] Blaisdell A. P. Stahlman W. D. Stolyarova A. (2016). The law of expect or a modified law of effect? Conductual 4, 61–90. doi: 10.59792/VEEC8896

[B24] Boakes R. A. (1977). “Performance on learning to associate a stimulus with positive reinforcement,” in Operant-Pavlovian Interactions, eds. H. David, and H. M. B. Hurwitz (Hillsdale, NJ: Erlbaum/Routledge), 67–101. doi: 10.4324/9781003150404-4

[B25] Bradfield L. A. Mahmoudi M. Wang E. Watson P. Panayi M. Rehn S. . (2026). The back-translation of value-modulated attentional capture from humans to rodents. J. Exper. Psychol. Anim. Learn. Cogn. doi: 10.1037/xan0000420 41701225

[B26] Brevers D. Koritzky G. Bechara A. Noël X. (2014). Cognitive processes underlying impaired decision-making under uncertainty in gambling disorder. Addict. Behav. 39, 1533–1536. doi: 10.1016/j.addbeh.2014.06.004 24980287 PMC4114505

[B27] Bromberg-Martin E. S. Monosov I. E. (2020). Neural circuitry of information seeking. Curr. Opin. Behav. Sci. 35, 62–70. doi: 10.1016/j.cobeha.2020.07.006 33681428 PMC7928425

[B28] Byrom N. C. Murphy R. A. (2018). Individual differences are more than a gene × environment interaction : the role of learning. J. Exper. Psychol. Anim. Learn. Cogn. 44, 36–55. doi: 10.1037/xan0000157 29323517

[B29] Cannon C. M. Bseikri M. R. (2004). Is dopamine required for natural reward? Physiol. Behav. 81, 741–748. doi: 10.1016/j.physbeh.2004.04.020 15234179

[B30] Cannon C. M. Palmiter R. D. (2003). Reward without dopamine. J. Neurosci. 23, 10827–10831. doi: 10.1523/JNEUROSCI.23-34-10827.2003 14645475 PMC6740991

[B31] Carrasco-Pujante J. Bringas C. Malaina I. Fedetz M. Martínez L. Pérez-Yarza G. . (2021). Associative conditioning is a robust systemic behavior in unicellular organisms: an interspecies comparison. Front. Microbiol. 12:707086. doi: 10.3389/fmicb.2021.707086 34349748 PMC8327096

[B32] Castner S. A. Goldman-Rakic P. S. (1999). Long-lasting psychotomimetic consequences of repeated low-dose amphetamine exposure in rhesus monkeys. Neuropsychopharmacology 20, 10–28. doi: 10.1016/S0893-133X(98)00050-5 9885781

[B33] Chelazzi L. Perlato A. Santandrea E. Della Libera C. (2013). Rewards teach visual selective attention. Vision Res. 85, 58–72. doi: 10.1016/j.visres.2012.12.005 23262054

[B34] Cherkasova M. V. Clark L. Barton J. J. S. Stoessl A. J. Winstanley C. A. (2024). Risk-promoting effects of reward-paired cues in human sign- and goal-trackers. Behav. Brain Res. 461:114865. doi: 10.1016/j.bbr.2024.114865 38220058

[B35] Cho S. A. Cho Y. S. (2021). Uncertainty modulates value-driven attentional capture. Atten. Percept. Psychophys. 83, 142–155. doi: 10.3758/s13414-020-02171-3 33155126

[B36] Chow J. J. Nickell J. R. Darna M. Beckmann J. S. (2016). Toward isolating the role of dopamine in the acquisition of incentive salience attribution. Neuropharmacology 109, 320–331. doi: 10.1016/j.neuropharm.2016.06.028 27371135 PMC4970875

[B37] Chow J. Y. L. Garner K. G. Pearson D. Heber J. Le Pelley M. E. (2025). Effects of instructed and experienced uncertainty on attentional priority. J. Exp. Psychol. Learn. Mem. Cogn. 51, 869–880. doi: 10.1037/xlm0001427 39680009

[B38] Clark L. Zack M. (2023). Engineered highs: reward variability and frequency as potential prerequisites of behavioural addiction. Addict. Behav. 140:107626. doi: 10.1016/j.addbeh.2023.107626 36701907

[B39] Colaizzi J. M. Flagel S. B. Gearhardt A. N. Borowitz M. A. Kuplicki R. Zotev V. . (2023). The propensity to sign-track is associated with externalizing behavior and distinct patterns of reward-related brain activation in youth. Sci. Rep. 13:4402. doi: 10.1038/s41598-023-30906-3 36928057 PMC10020483

[B40] Colaizzi J. M. Flagel S. B. Joyner M. A. Gearhardt A. N. Stewart J. L. Paulus M. P. (2020). Mapping sign-tracking and goal-tracking onto human behaviors. Neurosci. Biobehav. Rev. 111, 84–94. doi: 10.1016/j.neubiorev.2020.01.018 31972203 PMC8087151

[B41] Colom M. (2023). Neurobiological and Behavioural Studies of Individual Variation in Cue-Evoked Motivation Across Rodents And Humans (Ph.D. thesis). The Open University. Available online at: https://oro.open.ac.uk/90812/ (Accessed January 29, 2024).

[B42] Cope L. M. Gheidi A. Martz M. E. Duval E. R. Khalil H. Allerton T. . (2023). A mechanical task for measuring sign- and goal-tracking in humans: a proof-of-concept study. Behav. Brain Res. 436:114112. doi: 10.1016/j.bbr.2022.114112 36115435 PMC10153473

[B43] Crawford L. L. Steirn J. N. Pavlik W. B. (1985). Within- and between-subjects partial reinforcement effects with an autoshaped response using Japanese quail (Coturnix coturnix japonica). Anim. Learn. Behav. 13, 85–92. doi: 10.3758/BF03213369

[B44] Day J. J. Wheeler R. A. Roitman M. F. Carelli R. M. (2006). Nucleus accumbens neurons encode Pavlovian approach behaviors : evidence from an autoshaping paradigm. Eur. J. Neurosci. 23, 1341–1351. doi: 10.1111/j.1460-9568.2006.04654.x 16553795

[B45] Dayan P. Berridge K. C. (2014). Model-based and model-free Pavlovian reward learning: revaluation, revision, and revelation. Cogn. Affect. Behav. Neurosci. 14, 473–492. doi: 10.3758/s13415-014-0277-8 24647659 PMC4074442

[B46] de Jonge F. H. Ooms M. Kuurman W. W. Maes J. H. R. Spruijt B. M. (2008). Are pigs sensitive to variability in food rewards? Appl. Anim. Behav. Sci. 114, 93–104. doi: 10.1016/j.applanim.2008.01.004

[B47] Degni L. A. E. Danti C. Finotti G. Starita F. di Pellegrino G. Garofalo S. (2025). Pavlovian bias instigates suboptimal choices in humans. Behav. Res. Ther. 195:104906. doi: 10.1016/j.brat.2025.104906 41161143

[B48] Degni L. A. E. Mattioni L. Danti C. Bernardi V. Finotti G. Badioli M. . (2026). Reduced pavlovian value updating alters decision-making in sign-trackers. J. Neurosci. 46. doi: 10.1523/JNEUROSCI.1465-25.2025 41429483 PMC12828878

[B49] Della Libera C. Chelazzi L. (2006). Visual selective attention and the effects of monetary rewards. Psychol. Sci. 17, 222–227. doi: 10.1111/j.1467-9280.2006.01689.x 16507062

[B50] Della Libera C. Chelazzi L. (2009). Learning to attend and to ignore is a matter of gains and losses. Psychol. Sci. 20, 778–784. doi: 10.1111/j.1467-9280.2009.02360.x 19422618

[B51] Deluchi M. Costa F. S. Friedman R. Gonçalves R. Bizarro L. (2017). Attentional bias to unhealthy food in individuals with severe obesity and binge eating. Appetite 108, 471–476. doi: 10.1016/j.appet.2016.11.012 27836635

[B52] Dinu L.-M. Georgescu A.-L. Singh S. N. Byrom N. C. Overton P. G. Singer B. F. . (2024). Sign-tracking and goal-tracking in humans : utilising eye-tracking in clinical and non-clinical populations. Behav. Brain Res. 461:114846. doi: 10.1016/j.bbr.2024.114846 38184207

[B53] Dixon M. J. Harrigan K. A. Santesso D. L. Graydon C. Fugelsang J. A. Collins K. (2014). The impact of sound in modern multiline video slot machine play. J. Gambl. Stud. 30, 913–929. doi: 10.1007/s10899-013-9391-8 23821220 PMC4225056

[B54] Donoso J. R. Packheiser J. Pusch R. Lederer Z. Walther T. Uengoer M. . (2021). Emergence of complex dynamics of choice due to repeated exposures to extinction learning. Anim. Cogn. 24, 1279–1297. doi: 10.1007/s10071-021-01521-4 33978856 PMC8492564

[B55] Doran K. S. (2016). Translational Approaches to Studying Reward-Based Purposive Behaviours (Ph.D. thesis). University of Sussex. Available online at: https://sussex.figshare.com/articles/thesis/Translational_approaches_to_studying_reward-based_purposive_behaviours/23438579/1 (Accessed December 12, 2023).

[B56] Duckworth J. J. Wright H. Christiansen P. Rose A. K. Fallon N. (2022). Sign-tracking modulates reward-related neural activation to reward cues, but not reward feedback. Eur. J. Neurosci. 56, 5000–5013. doi: 10.1111/ejn.15787 35912531 PMC9804758

[B57] Easdale L. C. Le Pelley M. E. Beesley T. (2019). The onset of uncertainty facilitates the learning of new associations by increasing attention to cues. Q. J. Exp. Psychol. 72, 193–208. doi: 10.1080/17470218.2017.1363257 28766369

[B58] Eisenreich B. R. Hayden B. Y. Zimmermann J. (2019). Macaques are risk-averse in a freely moving foraging task. Sci. Rep. 9:15091. doi: 10.1038/s41598-019-51442-z 31636348 PMC6803699

[B59] Enkel T. Bartsch D. Bähner F. (2019). Sign- and goal-tracking rats show differences in various executive functions. Behav. Brain Res. 371:111979. doi: 10.1016/j.bbr.2019.111979 31141726

[B60] Esber G. R. Haselgrove M. (2011). Reconciling the influence of predictiveness and uncertainty on stimulus salience: a model of attention in associative learning. Proc. Biol. Sci. 278, 2553–2561. doi: 10.1098/rspb.2011.0836 21653585 PMC3136838

[B61] Everitt B. J. Dickinson A. Robbins T. W. (2001). The neuropsychological basis of addictive behaviour. Brain Res. Rev. 38, 36, 129–138. doi: 10.1016/S0165-0173(01)00088-1 11690609

[B62] Failing M. Theeuwes J. (2018). Selection history: how reward modulates selectivity of visual attention. Psychon. Bull. Rev. 25, 514–538. doi: 10.3758/s13423-017-1380-y 28986770 PMC5902518

[B63] Field M. Werthmann J. Franken I. Hofmann W. Hogarth L. Roefs A. (2016). The role of attentional bias in obesity and addiction. Health Psychol. 35, 767–780. doi: 10.1037/hea0000405 27505196

[B64] Fiorillo C. D. Tobler P. N. Schultz W. (2003). Discrete coding of reward probability and uncertainty by dopamine neurons. Science 299, 1898–1902. doi: 10.1126/science.1077349 12649484

[B65] Flagel S. B. Cameron C. Pickup K. Watson S. Robinson T. (2011a). A food predictive cue must be attributed with incentive salience for it to induce c-FOS mRNA expression in cortico-striatal-thalamic brain regions. Neuroscience 196, 80–96. doi: 10.1016/j.neuroscience.2011.09.004 21945724 PMC3206316

[B66] Flagel S. B. Clark J. J. Robinson T. E. Mayo L. Czuj A. Willuhn I. . (2011b). A selective role for dopamine in stimulus–reward learning. Nature 469, 53–57. doi: 10.1038/nature09588 21150898 PMC3058375

[B67] Flagel S. B. Robinson T. E. Clark J. J. Clinton S. M. Watson S. J. Seeman P. . (2010). An animal model of genetic vulnerability to behavioral disinhibition and responsiveness to reward-related cues: implications for addiction. Neuropsychopharmacology 35:2. doi: 10.1038/npp.2009.142 PMC2794950 19794408

[B68] Flagel S. B. Robinson T. E. Sarter M. (2021). Comment on Pohorala et al.: sign-tracking as a predictor of addiction vulnerability. Psychopharmacology 238, 2661–2664. doi: 10.1007/s00213-021-05927-3 34308488 PMC9248762

[B69] Flagel S. B. Watson S. J. Robinson T. E. Akil H. (2007). Individual differences in the propensity to approach signals vs goals promote different adaptations in the dopamine system of rats. Psychopharmacology 191, 599–607. doi: 10.1007/s00213-006-0535-8 16972103

[B70] Flayelle M. Brevers D. King D. L. Maurage P. Perales J. C. Billieux J. (2023). A taxonomy of technology design features that promote potentially addictive online behaviours. Nat. Rev. Psychol. 2, 136–150. doi: 10.1038/s44159-023-00153-4

[B71] Folk C. L. Anderson B. A. (2010). Target-uncertainty effects in attentional capture : color-singleton set or multiple attentional control settings? Psychon. Bull. Rev. 17, 421–426. doi: 10.3758/PBR.17.3.421 20551369

[B72] Frings C. Merz S. Hommel B. (2019). The impact of stimulus uncertainty on attentional control. Cognition 183, 208–212. doi: 10.1016/j.cognition.2018.10.017 30496911

[B73] Fuentes-Verdugo E. Pellón R. Papini M. R. Torres C. Fernández-Teruel A. Anselme P. (2020). Effects of partial reinforcement on autoshaping in inbred Roman high- and low-avoidance rats. Physiol. Behav. 225:113111. doi: 10.1016/j.physbeh.2020.113111 32738315

[B74] Garofalo S. di Pellegrino G. (2015). Individual differences in the influence of task-irrelevant Pavlovian cues on human behavior. Front. Behav. Neurosci. 9:163. doi: 10.3389/fnbeh.2015.00163 26157371 PMC4478391

[B75] Gibbon J. Farrell L. Locurto C. M. Duncan H. J. Terrace H. S. (1980). Partial reinforcement in autoshaping with pigeons. Anim. Learn. Behav. 8, 45–59. doi: 10.3758/BF03209729

[B76] Glueck A. C. Torres C. Papini M. R. (2018). Transfer between anticipatory and consummatory tasks involving reward loss. Learn. Motiv. 63, 105–125. doi: 10.1016/j.lmot.2018.05.001

[B77] Gneezy U. List J. A. Wu G. (2006). The uncertainty effect: when a risky prospect is valued less than its worst possible outcome. Q. J. Econ. 121, 1283–1309. doi: 10.1093/qje/121.4.1283

[B78] Gottlieb D. A. (2004). Acquisition with partial and continuous reinforcement in pigeon autoshaping. Learn. Behav. 32, 321–334. doi: 10.3758/BF03196031 15672827

[B79] Gottlieb D. A. (2005). Acquisition with partial and continuous reinforcement in rat magazine approach. J. Exp. Psychol. Anim. Behav. Process. 31, 319–333. doi: 10.1037/0097-7403.31.3.319 16045386

[B80] Gottlieb D. A. (2006). Effects of partial reinforcement and time between reinforced trials on terminal response rate in pigeon autoshaping. Behav. Processes, 72, 6–13. doi: 10.1016/j.beproc.2005.11.008 16413974

[B81] Gottlieb J. Oudeyer P.-Y. Lopes M. Baranes A. (2013). Information-seeking, curiosity, and attention: computational and neural mechanisms. Trends Cogn. Sci. 17, 585–593. doi: 10.1016/j.tics.2013.09.001 24126129 PMC4193662

[B82] Griffiths M. D. (1993). Fruit machine gambling: the importance of structural characteristics. J. Gambl. Stud. 9, 101–120. doi: 10.1007/BF01014863

[B83] Harris J. A. Carpenter J. S. (2011). Response rate and reinforcement rate in Pavlovian conditioning. J. Exp. Psychol. Anim. Behav. Process. 37, 375–384. doi: 10.1037/a0024554 21787098

[B84] Hart A. S. Clark J. J. Phillips P. E. M. (2015). Dynamic shaping of dopamine signals during probabilistic Pavlovian conditioning. Neurobiol. Learn. Mem. 117, 84–92. doi: 10.1016/j.nlm.2014.07.010 25172480 PMC4293327

[B85] Hearst E. Jenkins H. M. (1974). Sign-Tracking : The Stimulus-Reinforcer Relation and Directed Action. Austin, TX: Psychonomic Society.

[B86] Heck M. Durieux N. Anselme P. Quertemont E. (2025a). Implementations of sign- and goal-tracking behavior in humans: a scoping review. Cogn. Affect. Behav. Neurosci. 25, 263–290. doi: 10.3758/s13415-024-01230-8 39496905

[B87] Heck M. Simon J. Lesenfants D. Didone V. Anselme P. Quertemont E. (2025b). Sign tracking and alcohol consumption: a translational computerized task assessing individual differences in humans. Motiv. Sci. doi: 10.1037/mot0000408

[B88] Hellberg S. N. Russell T. I. Robinson M. J. F. (2019). Cued for risk: evidence for an incentive sensitization framework to explain the interplay between stress and anxiety, substance abuse, and reward uncertainty in disordered gambling behavior. Cogn. Affect. Behav. Neurosci. 19, 737–758. doi: 10.3758/s13415-018-00662-3 30357661 PMC6482104

[B89] Huys Q. J. M. Tobler P. N. Hasler G. Flagel S. B. (2014). The role of learning-related dopamine signals in addiction vulnerability. Prog. Brain Res. 211, 31–77. doi: 10.1016/B978-0-444-63425-2.00003-9 24968776

[B90] Iglesias A. G. Chiu A. S. Wong J. Campus P. Li F. Liu Z. (Nemo) Bhatti J. K. . (2023). Inhibition of dopamine neurons prevents incentive value encoding of a reward cue: with revelations from deep phenotyping. J. Neurosci. 43, 7376–7392. doi: 10.1523/JNEUROSCI.0848-23.2023 37709540 PMC10621773

[B91] Joyner M. (2019). Examining Behavioral Phenotypes of Overeating and Obesity: environmental, Psychological, and Neurobiological Influences on Food Motivation and Palatable Food Consumption (Ph.D. thesis). Available online at: http://deepblue.lib.umich.edu/handle/2027.42/151531 (Accessed January 29, 2024).

[B92] Ju J. Cho Y. S. (2023). The modulation of value-driven attentional capture by exploration for reward information. J. Exp. Psychol. Learn. Mem. Cogn. 49, 181–197. doi: 10.1037/xlm0001189 36265043

[B93] Kahneman D. (2011). Thinking, Fast and Slow. New York, NY: Farrar, Straus and Giroux, 499.

[B94] Kalhan S. Garrido M. I. Hester R. Redish A. D. (2023). Reward prediction-errors weighted by cue salience produces addictive behaviours in simulations, with asymmetrical learning and steeper delay discounting. Neural Netw. 168, 631–651. doi: 10.1016/j.neunet.2023.09.032 37844522

[B95] Kalhan S. Redish A. D. Hester R. Garrido M. I. (2021). A salience misattribution model for addictive-like behaviors. Neurosci. Biobehav. Rev. 125, 466–477. doi: 10.1016/j.neubiorev.2021.02.039 33657434 PMC8211366

[B96] Kaneko R. Simpson E. H. Balsam P. D. (2025). Impact of temporal uncertainty on sign-tracking behavior. J. Exper. Psychol. Anim. Learn. Cogn. 51, 103–111. doi: 10.1037/xan0000394 40193518 PMC12208561

[B97] Karlsson M. P. Tervo D. G. R. Karpova A. Y. (2012). Network resets in medial prefrontal cortex mark the onset of behavioral uncertainty. Science 338, 135–139. doi: 10.1126/science.1226518 23042898

[B98] Kawa A. B. Bentzley B. S. Robinson T. E. (2016). Less is more : prolonged intermittent access cocaine self-administration produces incentive-sensitization and addiction-like behavior. Psychopharmacology 233, 3587–3602. doi: 10.1007/s00213-016-4393-8 27481050 PMC5023484

[B99] Kindedji K. M. Huppé-Gourgues F. (2024). Quantification of attentional capacity and motivation in sign-tracking and goal-tracking behavior. Psychol. Neurosci. 17, 314–325. doi: 10.1037/pne0000346

[B100] Koshy Cherian A. Kucinski A. Pitchers K. Yegla B. Parikh V. Kim Y. . (2017). Unresponsive choline transporter as a trait neuromarker and a causal mediator of bottom-up attentional biases. J. Neurosci. 37, 2947–2959. doi: 10.1523/JNEUROSCI.3499-16.2017 28193693 PMC5354335

[B101] Krause M. A. Domjan M. (2017). “Ethological and evolutionary perspectives on Pavlovian conditioning,” in APA Handbook of Comparative Psychology: Perception, Learning, and Cognition, Vol. 2, eds. J. Call, G. M. Burghardt, I. M. Pepperberg, C. T. Snowdon, and T. Zentall (Washington, DC: American Psychological Association), 247–266. doi: 10.1037/0000012-012

[B102] Le Pelley M. E. Mitchell C. J. Beesley T. George D. N. Wills A. J. (2016). Attention and associative learning in humans: an integrative review. Psychol. Bull. 142, 1111–1140. doi: 10.1037/bul0000064 27504933

[B103] Le Pelley M. E. Pearson D. Griffiths O. Beesley T. (2015). When goals conflict with values: counterproductive attentional and oculomotor capture by reward-related stimuli. J. Exp. Psychol. Gen. 144, 158–171. doi: 10.1037/xge0000037 25420117

[B104] Le Pelley M. E. Pearson D. Porter A. Yee H. Luque D. (2019). Oculomotor capture is influenced by expected reward value but (maybe) not predictiveness. Q. J. Exp. Psychol. 72, 168–181. doi: 10.1080/17470218.2017.1313874 28375688

[B105] Le Pelley M. E. Watson P. Wiers R. W. (2024). Biased choice and incentive salience: implications for addiction. Behav. Neurosci. 138, 235–243. doi: 10.1037/bne0000583 38780585

[B106] Lee B. Gentry R. N. Bissonette G. B. Herman R. J. Mallon J. J. Bryden D. W. . (2018). Manipulating the revision of reward value during the intertrial interval increases sign tracking and dopamine release. PLoS Biol. 16:e2004015. doi: 10.1371/journal.pbio.2004015 30256785 PMC6175531

[B107] Lehner R. Balsters J. H. Bürgler A. Hare T. A. Wenderoth N. (2017). Food-predicting stimuli differentially influence eye movements and goal-directed behavior in normal-weight, overweight, and obese individuals. Front. Psychiatry 8:230. doi: 10.3389/fpsyt.2017.00230 29180968 PMC5693873

[B108] Lejuez C. W. Magidson J. F. Mitchell S. H. Sinha R. Stevens M. C. De Wit H. (2010). Behavioral and biological indicators of impulsivity in the development of alcohol use, problems, and disorders. Alcohol. Clin. Exp. Res. 34, 1334–1345. doi: 10.1111/j.1530-0277.2010.01217.x 20491733 PMC3182265

[B109] Lemos J. C. Wanat M. J. Smith J. S. Reyes B. A. S. Hollon N. G. Van Bockstaele E. J. . (2012). Severe stress switches CRF action in the nucleus accumbens from appetitive to aversive. Nature 490, 402–406. doi: 10.1038/nature11436 22992525 PMC3475726

[B110] Liu C. Yücel M. Suo C. Le Pelley M. E. Tiego J. Rotaru K. . (2021). Reward-related attentional capture moderates the association between fear-driven motives and heavy drinking. Eur. Addict. Res. 27, 351–361. doi: 10.1159/000513470 33706304

[B111] Lopez S. A. Mubarak E. Yang C. Parsegian A. Klumpner M. Campus P. . (2021). Male goal-tracker and sign-tracker rats do not differ in neuroendocrine or behavioral measures of stress reactivity. eNeuro 8:ENEURO.0384-20.2021. doi: 10.1523/ENEURO.0384-20.2021 33731330 PMC8116112

[B112] Lovic V. Saunders B. T. Yager L. M. Robinson T. E. (2011). Rats prone to attribute incentive salience to reward cues are also prone to impulsive action. Behav. Brain Res. 223, 255–261. doi: 10.1016/j.bbr.2011.04.006 21507334 PMC3119757

[B113] Mackintosh N. J. (1975). A theory of attention : variations in the associability of stimuli with reinforcement. Psychol. Rev. 82, 276–298. doi: 10.1037/h0076778

[B114] Mahmoudi S. Peck S. Madden G. J. (2023). Effects of inter-trial interval on sign-tracking and conditioned reinforcer efficacy in female rats. Behav. Processes 210:104911. doi: 10.1016/j.beproc.2023.104911 37406869 PMC10528028

[B115] Mascia P. Neugebauer N. M. Brown J. Bubula N. Nesbitt K. M. Kennedy R. T. . (2019). Exposure to conditions of uncertainty promotes the pursuit of amphetamine. Neuropsychopharmacology 44, 274–280. doi: 10.1038/s41386-018-0099-4 29875447 PMC6300556

[B116] Massa N. B. Crotty N. Levy I. Grubb M. A. (2024). Manipulating the reliability of target-color information modulates value-driven attentional capture. Atten. Percept. Psychophys. 86, 1108–1119. doi: 10.3758/s13414-024-02878-7 38538947 PMC11093855

[B117] Mechelmans D. J. Irvine M. Banca P. Porter L. Mitchell S. Mole T. B. . (2014). Enhanced attentional bias towards sexually explicit cues in individuals with and without compulsive sexual behaviours. PLoS One 9:e105476. doi: 10.1371/journal.pone.0105476 25153083 PMC4143289

[B118] Meyer P. J. Lovic V. Saunders B. T. Yager L. M. Flagel S. B. Morrow J. D. . (2012). Quantifying individual variation in the propensity to attribute incentive salience to reward cues. PLoS ONE 7:e38987. doi: 10.1371/journal.pone.0038987 22761718 PMC3382216

[B119] Mirenowicz J. Schultz W. (1994). Importance of unpredictability for reward responses in primate dopamine neurons. J. Neurophysiol. 72, 1024–1027. doi: 10.1152/jn.1994.72.2.1024 7983508

[B120] Moin Afshar N. Cinotti F. Martin D. Khamassi M. Calu D. J. Taylor J. R. . (2023). Reward-mediated, model-free reinforcement-learning mechanisms in pavlovian and instrumental tasks are related. J. Neurosci. 43, 458–471. doi: 10.1523/JNEUROSCI.1113-22.2022 36216504 PMC9864557

[B121] Monosov I. E. (2020). How outcome uncertainty mediates attention, learning, and decision-making. Trends Neurosci. 43, 795–809. doi: 10.1016/j.tins.2020.06.009 32736849 PMC8153236

[B122] Monosov I. E. (2024). Curiosity: primate neural circuits for novelty and information seeking. Nat. Rev. Neurosci. 25, 195–208. doi: 10.1038/s41583-023-00784-9 38263217

[B123] Morrison S. E. Bamkole M. A. Nicola S. M. (2015). Sign tracking, but not goal tracking, is resistant to outcome devaluation. Front. Neurosci. 9:468. doi: 10.3389/fnins.2015.00468 26733783 PMC4679928

[B124] Morriss J. Lee C. E. Wood A. Zhang J. Seabrooke T. (2024). Attentional bias to uncertainty-based information: a conceptual replication of Fergus et al. (2013). Curr. Psychol. 43, 24279–24286. doi: 10.1007/s12144-024-06067-5

[B125] Navarro V. Dwyer D. M. Honey R. C. (2024). Variation in the effectiveness of reinforcement and nonreinforcement in generating different conditioned behaviors. Neurobiol. Learn. Mem. 211:107915. doi: 10.1016/j.nlm.2024.107915 38527649

[B126] Ndiaye N. A. Shamleh S. A. Casale D. Castaneda-Ouellet S. Laplante I. Robinson M. J. F. . (2024). Relapse after intermittent access to cocaine: discriminative cues more effectively trigger drug seeking than do conditioned cues. Psychopharmacology 241, 2015–2032. doi: 10.1007/s00213-024-06614-9 38767684

[B127] Noseworthy T. J. Finlay K. (2009). A comparison of ambient casino sound and music: effects on dissociation and on perceptions of elapsed time while playing slot machines. J. Gambl. Stud. 25, 331–342. doi: 10.1007/s10899-009-9136-x 19582553

[B128] O'Reilly J. X. Schüffelgen U. Cuell S. F. Behrens T. E. J. Mars R. B. Rushworth M. F. S. (2013). Dissociable effects of surprise and model update in parietal and anterior cingulate cortex. Proc. Nat. Acad. Sci. 110, E3660–E3669. doi: 10.1073/pnas.1305373110 23986499 PMC3780876

[B129] Palmiter R. D. (2008). Dopamine signaling in the dorsal striatum is essential for motivated behaviors: lessons from dopamine-deficient mice. Ann. N. Y. Acad. Sci. 1129, 35–46. doi: 10.1196/annals.1417.003 18591467 PMC2720267

[B130] Paolone G. Angelakos C. C. Meyer P. J. Robinson T. E. Sarter M. (2013). Cholinergic control over attention in rats prone to attribute incentive salience to reward cues. J. Neurosci. 33, 8321–8335. doi: 10.1523/JNEUROSCI.0709-13.2013 23658172 PMC3690461

[B131] Parke J. Griffiths M. (2006). The psychology of the fruit machine: the role of structural characteristics (revisited). Int. J. Ment. Health Addict. 4, 151–179. doi: 10.1007/s11469-006-9014-z

[B132] Paulson P. E. Robinson T. E. (1995). Amphetamine-induced time-dependent sensitization of dopamine neurotransmission in the dorsal and ventral striatum : a microdialysis study in behaving rats. Synapse 19, 56–65. doi: 10.1002/syn.890190108 7709344 PMC1859849

[B133] Pavlov I. P. (1927). Conditioned Reflexes: An Investigation of the Physiological Activity of the Cerebral Cortex. London: Oxford University Press, xv, 430. 10.5214/ans.0972-7531.1017309 PMC4116985 25205891

[B134] Pearce J. M. Hall G. (1980). A model for Pavlovian learning : variations in the effectiveness of conditioned but not of unconditioned stimuli. Psychol. Rev. 87, 532–552. doi: 10.1037/0033-295X.87.6.532 7443916

[B135] Pearce J. M. Kaye H. Collins L. (1985). A comparison of the effects of partial reinforcement schedules using a within-subject serial autoshaping procedure. Q. J. Exp. Psychol. B 37, 379–396. doi: 10.1080/14640748508401176

[B136] Pearson D. Chong A. Chow J. Y. L. Garner K. G. Theeuwes J. Le Pelley M. E. (2024). Uncertainty-modulated attentional capture : outcome variance increases attentional priority. J. Exp. Psychol. Gen. 153, 1628–1643. doi: 10.1037/xge0001586 38695800

[B137] Pearson D. Donkin C. Tran S. C. Most S. B. Le Pelley M. E. (2015). Cognitive control and counterproductive oculomotor capture by reward-related stimuli. Vis. Cogn. 23, 41–66. doi: 10.1080/13506285.2014.994252

[B138] Peciña S. Cagniard B. Berridge K. C. Aldridge J. W. Zhuang X. (2003). Hyperdopaminergic mutant mice have higher “wanting” but not “liking” for sweet rewards. J. Neurosci. 23, 9395–9402. doi: 10.1523/JNEUROSCI.23-28-09395.2003 14561867 PMC6740586

[B139] Pellón R. Íbias J. Killeen P. R. (2018). Delay gradients for spout-licking and magazine-entering induced by a periodic food schedule. Psychol. Rec. 68, 151–162. doi: 10.1007/s40732-018-0275-2

[B140] Pitchers K. Phillips K. Jones J. Robinson T. Sarter M. (2017). Diverse roads to relapse : a discriminative cue signaling cocaine availability is more effective in renewing cocaine seeking in goal trackers than sign trackers and depends on basal forebrain cholinergic activity. J. Neurosci. 37, 0990–17. doi: 10.1523/JNEUROSCI.0990-17.2017 PMC5546399 28659281

[B141] Pitchford B. Arnell K. M. (2025). An examination of how reward associations facilitate and impair Stroop performance. Psychol. Res. 89:105. doi: 10.1007/s00426-025-02135-y 40439745

[B142] Preuschoff K. Bossaerts P. (2007). Adding prediction risk to the theory of reward learning. Ann. N. Y. Acad. Sci. 1104, 135–146. doi: 10.1196/annals.1390.005 17344526

[B143] Redish A. D. (2004). Addiction as a computational process gone awry. Science 306, 1944–1947. doi: 10.1126/science.1102384 15591205

[B144] Robinson M. J. F. Anselme P. (2019). How uncertainty sensitizes dopamine neurons and invigorates amphetamine-related behaviors. Neuropsychopharmacology 44, 237–238. doi: 10.1038/s41386-018-0130-9 29959440 PMC6300523

[B145] Robinson M. J. F. Anselme P. Fischer A. M. Berridge K. C. (2014). Initial uncertainty in Pavlovian reward prediction persistently elevates incentive salience and extends sign-tracking to normally unattractive cues. Behav. Brain Res. 266, 119–130. doi: 10.1016/j.bbr.2014.03.004 24631397 PMC4016791

[B146] Robinson M. J. F. Anselme P. Suchomel K. Berridge K. C. (2015). Amphetamine-induced sensitization and reward uncertainty similarly enhance incentive salience for conditioned cues. Behav. Neurosci. 129, 502–511. doi: 10.1037/bne0000064 26076340 PMC4540329

[B147] Robinson M. J. F. Berridge K. C. (2013). Instant transformation of learned repulsion into motivational “wanting”. Curr. Biol. 23, 282–289. doi: 10.1016/j.cub.2013.01.016 23375893 PMC3580026

[B148] Robinson M. J. F. Bonmariage Q. S. A. Samaha A.-N. (2023). Unpredictable, intermittent access to sucrose or water promotes increased reward pursuit in rats. Behav. Brain Res. 453:114612. doi: 10.1016/j.bbr.2023.114612 37544370

[B149] Robinson T. E. Berridge K. C. (1993). The neural basis of drug craving : an incentive-sensitization theory of addiction. Brain Res. Rev. 18, 247–291. doi: 10.1016/0165-0173(93)90013-P 8401595

[B150] Robinson T. E. Berridge K. C. (2025). The incentive-sensitization theory of addiction 30 years on. Annu. Rev. Psychol. 76, 29–58. doi: 10.1146/annurev-psych-011624-024031 39094061 PMC11773642

[B151] Robinson T. E. Carr C. Kawa A. B. (2018). “The propensity to attribute incentive salience to drug cues and poor cognitive control combine to render sign-trackers susceptible to addiction,” in Sign-Tracking and Drug Addiction, eds. A. Tomie, and J. Morrow (Ann Arbor, MI: Michigan Publishing, University of Michigan Library).

[B152] Robinson T. E. Flagel S. B. (2009). Dissociating the predictive and incentive motivational properties of reward-related cues through the study of individual differences. Biol. Psychiatry 65, 869–873. doi: 10.1016/j.biopsych.2008.09.006 18930184 PMC2737368

[B153] Robinson T. E. Yager L. M. Cogan E. S. Saunders B. T. (2014). On the motivational properties of reward cues : individual differences. Neuropharmacology 76(Pt B), 450–459. doi: 10.1016/j.neuropharm.2013.05.040 23748094 PMC3796005

[B154] Romero-Sosa J. L. Yeghikian A. Wikenheiser A. M. Blair H. T. Izquierdo A. (2025). Neural coding of choice and outcome are modulated by uncertainty in orbitofrontal but not secondary motor cortex. Nat. Commun. 16:8931. doi: 10.1038/s41467-025-63866-5 41062460 PMC12508120

[B155] Rosas J. M. García-Gutiérrez A. Callejas-Aguilera J. E. (2006). Effects of context change upon retrieval of first and second-learned information in human predictive learning. Psicológica 27, 35–56.

[B156] Sarter M. Bruno J. P. (1999). Abnormal regulation of corticopetal cholinergic neurons and impaired information processing in neuropsychiatric disorders. Trends Neurosci. 22, 67–74. doi: 10.1016/S0166-2236(98)01289-2 10092046

[B157] Sarter M. Gehring W. J. Kozak R. (2006). More attention must be paid : the neurobiology of attentional effort. Brain Res. Rev. 51, 145–160. doi: 10.1016/j.brainresrev.2005.11.002 16530842

[B158] Sarter M. Phillips K. B. (2018). The neuroscience of cognitive-motivational styles : sign- and goal-trackers as animal models. Behav. Neurosci. 132, 1–12. doi: 10.1037/bne0000226 29355335 PMC5881169

[B159] Saunders B. T. Robinson T. E. (2011). Individual Variation in the Motivational Properties of Cocaine. Neuropsychopharmacology 36, 1668–1676. doi: 10.1038/npp.2011.48 21471956 PMC3138662

[B160] Saunders B. T. Robinson T. E. (2012). The role of dopamine in the accumbens core in the expression of Pavlovian-conditioned responses. Eur. J. Neurosci. 36, 2521–2532. doi: 10.1111/j.1460-9568.2012.08217.x 22780554 PMC3424374

[B161] Saunders B. T. Yager L. M. Robinson T. E. (2013). Cue-evoked cocaine “craving”: role of dopamine in the accumbens core. J. Neurosci. 33, 13989–14000. doi: 10.1523/JNEUROSCI.0450-13.2013 23986236 PMC3756749

[B162] Schad D. J. Rapp M. A. Garbusow M. Nebe S. Sebold M. Obst E. . (2020). Dissociating neural learning signals in human sign- and goal-trackers. Nat. Hum. Behav. 4, 201–214. doi: 10.1038/s41562-019-0765-5 31712764

[B163] Schettino M. Mauti M. Parrillo C. Ceccarelli I. Giove F. Napolitano A. . (2024). Resting-state brain activation patterns and network topology distinguish human sign and goal trackers. Transl. Psychiatry 14, 1–10. doi: 10.1038/s41398-024-03162-w 39438457 PMC11496639

[B164] Schultz W. (1998). Predictive reward signal of dopamine neurons. J. Neurophysiol. 80, 1–27. doi: 10.1152/jn.1998.80.1.1 9658025

[B165] Schultz W. Apicella P. Ljungberg T. (1993). Responses of monkey dopamine neurons to reward and conditioned stimuli during successive steps of learning a delayed response task. J. Neurosci. 13, 900–913. doi: 10.1523/JNEUROSCI.13-03-00900.1993 8441015 PMC6576600

[B166] Schultz W. Dayan P. Montague P. R. (1997). A neural substrate of prediction and reward. Science 275, 1593–1599. doi: 10.1126/science.275.5306.1593 9054347

[B167] Shahan T. A. (2022). A theory of the extinction burst. Perspect. Behav. Sci. 45, 495–519. doi: 10.1007/s40614-022-00340-3 36249175 PMC9458838

[B168] Singer B. F. Anselme P. Robinson M. J. F. Vezina P. (2020). An overview of commonalities in the mechanisms underlying gambling and substance use disorders. Prog. Neuropsychopharmacol. Biol. Psychiatry 101:109944. doi: 10.1016/j.pnpbp.2020.109944 32289336

[B169] Singer B. F. Scott-Railton J. Vezina P. (2012). Unpredictable saccharin reinforcement enhances locomotor responding to amphetamine. Behav. Brain Res. 226, 340–344. doi: 10.1016/j.bbr.2011.09.003 21924296 PMC3216637

[B170] Sklenarik S. Potenza M. N. Gola M. Astur R. S. (2020). Approach bias for erotic stimuli among heterosexual female college students who use pornography. Addict. Behav. 108:106438. doi: 10.1016/j.addbeh.2020.106438 32325387

[B171] Soltani A. Izquierdo A. (2019). Adaptive learning under expected and unexpected uncertainty. Nat. Rev. Neurosci. 20, 635–644. doi: 10.1038/s41583-019-0180-y 31147631 PMC6752962

[B172] Starkweather C. K. Uchida N. (2021). Dopamine signals as temporal difference errors: recent advances. Curr. Opin. Neurobiol. 67, 95–105. doi: 10.1016/j.conb.2020.08.014 33186815 PMC8107188

[B173] Strand P. S. Robinson M. J. F. Fiedler K. R. Learn R. Anselme P. (2022). Quantifying the instrumental and noninstrumental underpinnings of Pavlovian responding with the Price equation. Psychon. Bull. Rev. 29, 1295–1306. doi: 10.3758/s13423-021-02047-z 34918283

[B174] Swintosky M. Brennan J. T. Koziel C. Paulus J. P. Morrison S. E. (2021). Sign tracking predicts suboptimal behavior in a rodent gambling task. Psychopharmacology 238, 2645–2660. doi: 10.1007/s00213-021-05887-8 34191111 PMC8500220

[B175] Theeuwes J. (2019). Goal-driven, stimulus-driven, and history-driven selection. Curr. Opin. Psychol. 29, 97–101. doi: 10.1016/j.copsyc.2018.12.024 30711911

[B176] Thériault N. Bourque M. J. Huppé-Gourgues F. (2025). The effects of multimodal distractors on sign-trackers and goal-trackers attention. Behav. Brain Res. 495:115800. doi: 10.1016/j.bbr.2025.115800 40915568

[B177] Tindell A. J. Smith K. S. Berridge K. C. Aldridge J. W. (2009). Dynamic computation of incentive salience: “wanting” what was never “liked”. J. Neurosci. 29, 12220–12228. doi: 10.1523/JNEUROSCI.2499-09.2009 19793980 PMC2792765

[B178] Toates F. M. (1986). Motivational Systems. Available online at: https://oro.open.ac.uk/66410/

[B179] Tobler P. N. O'doherty J. P. Dolan R. J. Schultz W. (2006). Human neural learning depends on reward prediction errors in the blocking paradigm. J. Neurophysiol. 95, 301–310. doi: 10.1152/jn.00762.2005 16192329 PMC2637603

[B180] Tomie A. Aguado A. S. Pohorecky L. A. Benjamin D. (1998). Ethanol induces impulsive-like responding in a delay-of-reward operant choice procedure: impulsivity predicts autoshaping. Psychopharmacology 139, 376–382. doi: 10.1007/s002130050728 9809858

[B181] Tomie A. Grimes K. L. Pohorecky L. A. (2008). Behavioral characteristics and neurobiological substrates shared by Pavlovian sign-tracking and drug abuse. Brain Res. Rev. 58, 121–135. doi: 10.1016/j.brainresrev.2007.12.003 18234349 PMC2582385

[B182] Trick L. Hogarth L. Duka T. (2011). Prediction and uncertainty in human Pavlovian to instrumental transfer. J. Exp. Psychol. Learn. Mem. Cogn. 37, 757–765. doi: 10.1037/a0022310 21319920

[B183] Tunstall B. J. Kearns D. N. (2015). Sign-tracking predicts increased choice of cocaine over food in rats. Behav. Brain Res. 281, 222–228. doi: 10.1016/j.bbr.2014.12.034 25541036 PMC4305489

[B184] van Holst R. J. van Sescousse G. Janssen L. K. Janssen M. Berry A. S. Jagust W. J. Cools R. (2018). Increased striatal dopamine synthesis capacity in gambling addiction. Biol. Psychiatry, 83, 1036–1043. doi: 10.1016/j.biopsych.2017.06.010 28728675 PMC6698370

[B185] Varazzani C. San-Galli A. Gilardeau S. Bouret S. (2015). Noradrenaline and dopamine neurons in the reward/effort trade-off: a direct electrophysiological comparison in behaving monkeys. J. Neurosci. 35, 7866–7877. doi: 10.1523/JNEUROSCI.0454-15.2015 25995472 PMC6795183

[B186] Verdejo-Garcia A. Albein-Urios N. (2021). Impulsivity traits and neurocognitive mechanisms conferring vulnerability to substance use disorders. Neuropharmacology 183:108402. doi: 10.1016/j.neuropharm.2020.108402 33189766

[B187] Versaggi C. L. King C. P. Meyer P. J. (2016). The tendency to sign-track predicts cue-induced reinstatement during nicotine self-administration, and is enhanced by nicotine but not ethanol. Psychopharmacology 233, 2985–2997. doi: 10.1007/s00213-016-4341-7 27282365 PMC4935618

[B188] Warlow S. M. Naffziger E. E. Berridge K. C. (2020). The central amygdala recruits mesocorticolimbic circuitry for pursuit of reward or pain. Nat. Commun. 11:2716. doi: 10.1038/s41467-020-16407-1 32483118 PMC7264246

[B189] Watson P. Prior K. Ridley N. Monds L. Manning V. Wiers R. W. . (2024). Sign-tracking to non-drug reward is related to severity of alcohol-use problems in a sample of individuals seeking treatment. Addict. Behav. 154:108010. doi: 10.1016/j.addbeh.2024.108010 38479081

[B190] White B. Clark A. Miller M. (2024). Digital being: social media and the predictive mind. Neurosci. Conscious. 2024:niae008. doi: 10.1093/nc/niae008 38504826 PMC10949958

[B191] Wiers R. W. Stacy A. W. (2006). Implicit cognition and addiction. Curr. Dir. Psychol. Sci. 15, 292–296. doi: 10.1111/j.1467-8721.2006.00455.x

[B192] Wiers R. W. van Gaal S. Le Pelley M. E. (2021). “Akrasia and addiction: neurophilosophy and psychological mechanisms,” in Social Neuroeconomics: Mechanistic Integration of the Neurosciences and the Social Sciences, eds. J. Harbecke, and C. Herrmann-Pillath (New York, NY: Routledge/Taylor and Francis Group), 121–147. doi: 10.4324/9780429296918-10

[B193] Winkielman P. Berridge K. (2003). Irrational wanting and subrational liking: how rudimentary motivational and affective processes shape preferences and choices. Polit. Psychol. 24, 657–680. doi: 10.1046/j.1467-9221.2003.00346.x

[B194] Witteman J. Post H. Tarvainen M. de Bruijn A. Perna E. D. S. F. Ramaekers J. G. . (2015). Cue reactivity and its relation to craving and relapse in alcohol dependence: a combined laboratory and field study. Psychopharmacology 232, 3685–3696. doi: 10.1007/s00213-015-4027-6 26257163 PMC4562995

[B195] Wittmann M. K. Kolling N. Akaishi R. Chau B. K. H. Brown J. W. Nelissen N. . (2016). Predictive decision making driven by multiple time-linked reward representations in the anterior cingulate cortex. Nat. Commun. 7:12327. doi: 10.1038/ncomms12327 27477632 PMC4974652

[B196] Yager L. M. Pitchers K. K. Flagel S. B. Robinson T. E. (2015). Individual variation in the motivational and neurobiological effects of an opioid cue. Neuropsychopharmacology 40, 1269–1277. doi: 10.1038/npp.2014.314 25425322 PMC4367472

[B197] Yokum S. Ng J. Stice E. (2011). Attentional bias to food images associated with elevated weight and future weight gain: an fMRI study. Obesity 19, 1775–1783. doi: 10.1038/oby.2011.168 21681221 PMC4007087

[B198] Zack M. Featherstone R. E. Mathewson S. Fletcher P. J. (2014). Chronic exposure to a gambling-like schedule of reward predictive stimuli can promote sensitization to amphetamine in rats. Front. Behav. Neurosci. 8:36. doi: 10.3389/fnbeh.2014.00036 24574987 PMC3920462

[B199] Zack M. St. George R. Clark L. (2020). Dopaminergic signaling of uncertainty and the aetiology of gambling addiction. Prog. Neuropsychopharmacol. Biol. Psychiatry 99:109853. doi: 10.1016/j.pnpbp.2019.109853 31870708

[B200] Zhang J. Berridge K. C. Tindell A. J. Smith K. S. Aldridge J. W. (2009). A neural computational model of incentive salience. PLoS Comput. Biol. 5:e1000437. doi: 10.1371/journal.pcbi.1000437 19609350 PMC2703828

